# Neural sequences underlying directed turning in *Caenorhabditis* *elegans*

**DOI:** 10.1038/s41593-026-02257-5

**Published:** 2026-04-10

**Authors:** Talya S. Kramer, Flossie K. Wan, Sarah M. Pugliese, Adam A. Atanas, Sreeparna Pradhan, Alex W. Hiser, Lillie M. Godinez, Jinyue Luo, Eric Bueno, Thomas Felt, Steven W. Flavell

**Affiliations:** 1 Howard Hughes Medical Institute, Picower Institute for Learning and Memory, Department of Brain & Cognitive Sciences, Massachusetts Institute of Technology, Cambridge, MA USA; 2 Department of Biology, Massachusetts Institute of Technology, Cambridge, MA USA

**Keywords:** Sensorimotor processing, Neural circuits

## Abstract

Complex behaviors, such as navigation, rely on sequenced motor outputs that combine to generate effective movement. The brain-wide organization of the circuits that integrate sensory signals to select appropriate motor sequences remains poorly understood. Here we characterize the architecture of neural circuits that control *Caenorhabditis* *elegans* olfactory navigation. We identify error-correcting turns during navigation and use whole-brain calcium imaging and cell-specific perturbations to determine their neural underpinnings. These turns occur as motor sequences accompanied by neural sequences, in which defined neurons activate in a stereotyped order during each turn. Distinct neurons in this sequence respond to the spatial distribution of attractive and aversive olfactory cues, anticipate upcoming turn directions and drive movement, linking key features of this sensorimotor behavior across time. The neuromodulator tyramine coordinates these sequential brain dynamics. Our results illustrate how neuromodulation can act on a defined neural architecture to link sensory cues to motor actions.

## Abstract (web summary)

During olfactory navigation, *Caenorhabditis* *elegans* can execute error-correcting turns. Whole-brain imaging and perturbation experiments identify an ensemble of neurons that control reorientation and turning behaviors.

## Main

Whether moving toward a food source or away from a predator, animals must integrate sensory stimuli to navigate to favorable locations. The neural circuits for navigation are tasked with generating directed movement while simultaneously integrating dynamic sensory input to continually update behavior. Understanding how neural circuits select, execute and update sensory-guided navigation behaviors should reveal basic principles of how nervous systems are organized to integrate sensory information and control behavior.

The neural circuits that control navigation need to relate the spatial distribution of sensory cues in the environment to an animal’s own movement. In mammals, neural representations of an animal’s location and movement patterns can be found in the hippocampus and surrounding structures^1^. In *Drosophila* and other arthropods, the central complex stores information about an animal’s heading direction^2^, which can be updated based on sensory cues^3^ to direct navigation^4,5^. Across species, navigation circuits are anatomically separated from motor circuits^6,7^. Understanding how these distributed neural circuits interact in the context of sensory navigation remains a key challenge.

Studying sensory-guided behavior is particularly tractable in *Caenorhabditis* *elegans*, which has robust behavioral responses to stimuli like odors, temperature, gases and salts^8^. The primary sensory neurons that respond to many stimuli have been identified^9^, and the neuronal connectome is defined for *C. elegans’* 302 neurons^10–12^. Additionally, brain-wide calcium imaging in freely moving animals^13,14^ with reliable neuronal identification^15–19^ has recently made it feasible to map brain-wide neural activity during specific *C. elegans* behaviors.

*C. elegans* olfactory navigation is a well-studied, naturalistic behavior. Animals have been thought to navigate using two behavioral strategies. First, a ‘biased random walk’, in which animals moving in an unfavorable sensory direction increase their reorientation rates^20–24^. *C. elegans* ‘reorientations’ are stereotyped behavioral sequences—animals switch to reverse movement for several seconds (‘reversals’) and then bend their heads to make a dorsal or ventral head bend (‘turn’) as they resume forward movement. Individual reorientations are hypothesized to be randomly directed, but reorientations often occur in clusters, termed ‘pirouettes’, which may allow an animal to sample until it finds a favorable direction^20,25,26^. Second, animals moving forward ‘weathervane’, bending their forward movement in a favorable direction^23^, including when they encounter sharp gradients^21^. Key interneurons required for biased random walk and weathervaning have been identified^22–24,27^. However, there is still a gap in our understanding of how ongoing neural dynamics across the entire system are coordinated to generate precisely sequenced behaviors.

Here we examine the neural circuits underlying olfactory navigation. First, we identify a new behavioral strategy during *C. elegans* olfactory navigation—animals modulate the angles of their individual reorientations based on the olfactory gradient, suggesting that they can compute their heading error in the gradient and perform error-correcting turns. Next, we use whole-brain calcium imaging and cell-specific perturbations to identify neurons that activate in a stereotyped sequence during each turn. These neurons respond to the spatial locations of attractive and aversive olfactory cues, bias upcoming turn angles, terminate reversals and execute turn kinematics. We also determine that the neuromodulator tyramine is critical for these circuit dynamics during each turn. These results suggest that ongoing neuromodulation and coordinated sequences of neural activity can link sensory signals to motor actions and facilitate sensory-guided movement.

## Results

### *C. elegans* directs the angles of its reorientations to improve its bearing during olfactory navigation

**Fig. 1:**
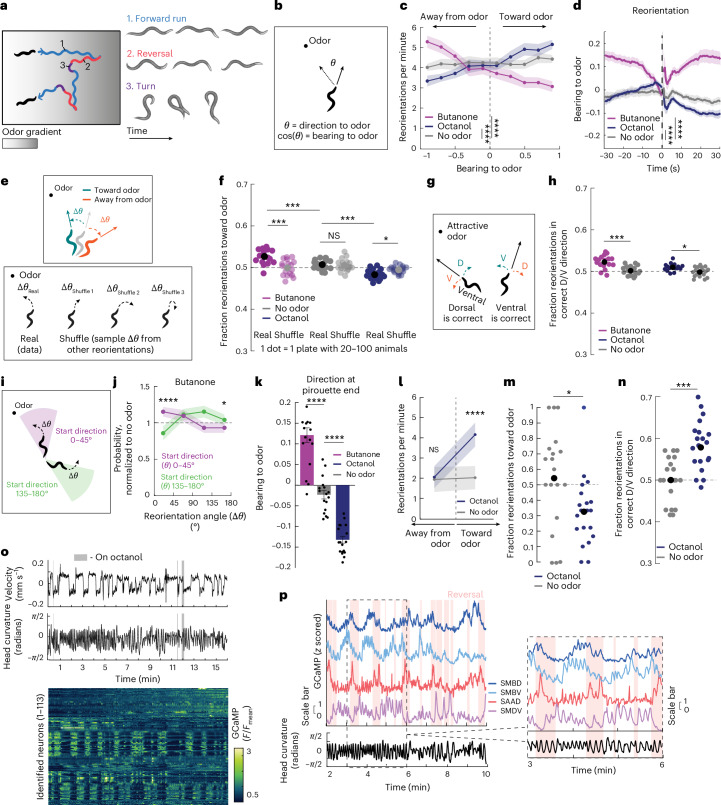
*C. elegans* directs the angles of its reorientations during olfactory navigation. **a**, Mock path of an animal navigating an odor gradient. Right, images showing corresponding animal postures. **b**, *θ* is the angle between the animal’s direction of movement (black arrow) and the point source of the odor (dashed line). Bearing to the odor is defined as cos(*θ*). **c**, Reorientation rate versus the animal’s bearing to odor (cos(*θ*)). Two-sided Wilcoxon rank-sum test with Bonferroni correction comparing slopes of the reorientation rate to no odor (butanone, *P* < 0.0001; octanol, *P* < 0.001). *n* = 17–18 recorded plates with 20–100 animals per plate. Data are means, shaded regions show 95% CI. **d**, Average bearing to odor over time, aligned to the onsets of reorientations (dashed line). Data during the reversal are removed. Two-sided Wilcoxon rank-sum test comparing the prereversal slopes of the bearing over time (butanone, *P* < 0.0001; octanol, *P* < 0.0001). *n* = 17–18 recorded plates. Data are mean ± 95% CI. **e**, Top, when animals reorient, they can turn toward (teal) or away from the odor (orange). Bottom, we combined animals’ real initial directions (*θ*) with randomly shuffled changes in direction (∆*θ*) sampled from other reorientations in the same video. Each initial angle is combined with one change in direction; three examples are shown. **f**, The fraction of the reorientations that turn the animal toward the odor in real and randomly shuffled data. Two-sided Wilcoxon rank-sum test with Bonferroni correction (from left to right, *P* = 0.0002, *P* = 0.0009, *P* = 0.6054, *P* < 0.0001, *P* = 0.0036). *n* = 17–18 recorded plates. Each dot is one plate with 20–100 animals; the black or gray dot shows data mean. **g**, Animals can direct reorientations dorsally or ventrally. Which choice is ‘correct’ depends on the initial bearing to odor. **h**, Fraction of reorientations that turn the animal in the correct dorsal or ventral direction. Each dot is one plate with 20–100 animals. Two-sided Wilcoxon rank-sum test (butanone, *P* = 0.0002; octanol, *P* = 0.0079). *n* = 17–18 recorded plates. Black dot shows data mean. **i**, Animals begin reorientations with a range of initial angles of their direction to the odor (*θ*). **j**, Change in direction (∆*θ*) during reorientations by animals that start with either large or small angle directions to the odor (*θ*), normalized to no-odor controls. Two-sided Wilcoxon rank-sum test with Bonferroni correction (from left to right, *P* < 0.0001, *P* = 0.0142). *n* = 18 recorded plates. Data show mean ± 95% CI. **k**, Bearing to odor at the ends of pirouettes. Two-sided Wilcoxon rank-sum test with Bonferroni correction (both *P* < 0.0001). *n* = 17–18 recorded plates. Data are mean ± s.e.m. **l**, Results from the sharp odor gradient. Reorientation rate in the 10 s after animals cross onto octanol (‘toward odor’) or cross from octanol to baseline agar (‘away from odor’). Dots show the mean reorientation rate across recordings. Two-sided Wilcoxon rank-sum test with Bonferroni correction (from left to right, *P* = 0.6651, *P* < 0.0001). *n* = 20 recording plates per condition. Data are mean ± 95% CI. **m**, Fraction of reorientations that occur at the agar boundary that ends with the animal facing toward the odor. Each dot indicates the fraction from one plate with five to ten worms. Two-sided Wilcoxon rank-sum test (*P* = 0.0201). *n* = 20 recordings per condition. Black dot shows data mean. **n**, Fraction of reorientations that occur at the agar boundary that turns the animal in the correct D/V direction (Methods). Each dot indicates the fraction from one plate with five to ten worms. Two-sided Wilcoxon rank-sum test (*P* = 0.0003). *n* = 20 recordings per condition. Black dots show data means. **o**, Sample brain-wide imaging dataset. Heatmap shows calcium traces (*F*/*F*_mean_) of 113 identified neurons. Velocity and head curvature are shown above. **p**, Traces of identified neurons from the same dataset as in **o**. CI, confidence interval; NS, not significant. For all panels, significance is noted as: NS (not significant), *P < 0.05, **P < 0.01, ***P < 0.001, ****P < 0.0001. For panels with multiple comparisons, symbols denote Bonferroni-adjusted *P* values.

**Extended Data Fig. 1:**
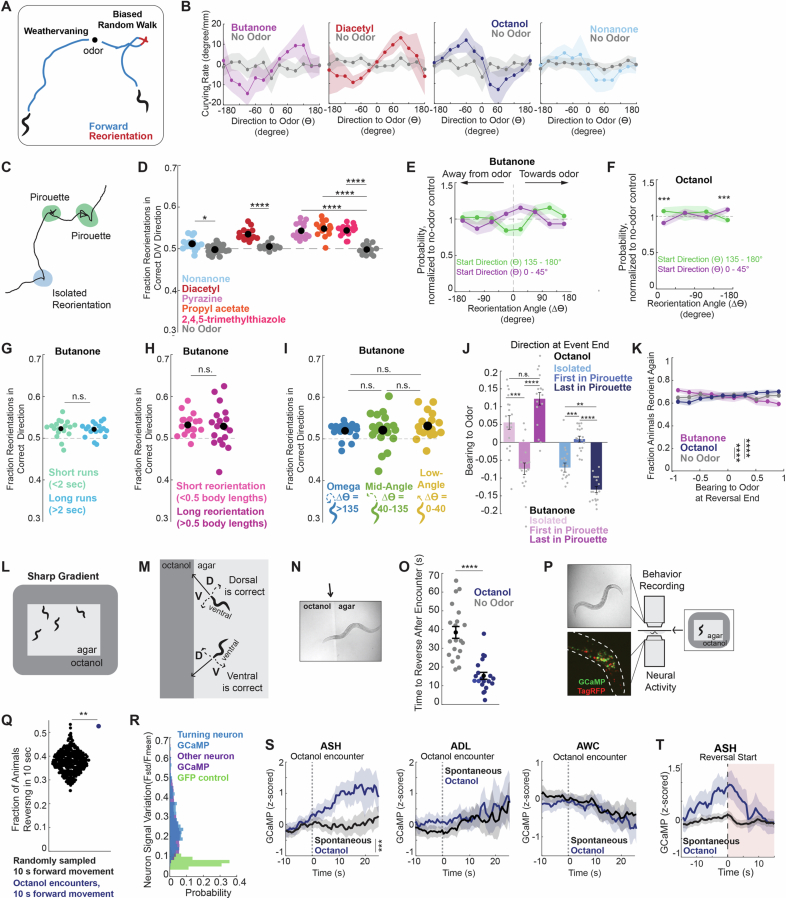
Further analysis of *C. elegans* olfactory navigation behavior. **a**, Visualization of the two main chemotaxis strategies: weathervaning (left) and biased random walk (right). Animals weathervane by bending their direction of forward movement in a favorable direction, either toward the attractive odor or away from the aversive odor^23^. Animals execute a biased random walk by increasing the likelihood of initiating reorientations (red) when they are moving in an unfavorable direction in the odor gradient (forward movement shown in blue)^20^. **b**, Weathervaning behavior in olfactory gradients. To test for the presence of weathervaning, we examined the curving rate of forward movement when animals had different directions to the odor (*θ*). Curving rate is the change in the animal’s heading divided by their change in displacement over 1 s, which can be thought of as a measure of how much and which way animals are bending forward runs (see Methods for further details). As previously shown^23^, animals bend runs toward attractive odors (butanone and diacetyl, sign of *θ* is the same as the sign of the curving rate). We further saw evidence that animals weathervane away from aversive odors (octanol and nonanone, sign of the curving is the opposite of the sign of *θ*). Data show mean ± 95% CI. **c**, Example animal movement path during chemotaxis showing a single, isolated reorientation (blue) and two examples of repeated reorientations that form pirouettes (green). **d**, Fraction of reorientations that turn the animal in the correct dorsal or ventral direction, comparing with odor to no odor. The diacetyl and nonanone with odor plates were recorded on different days, so each has its own no odor control, while 10 mg ml^−1^ pyrazine, 1:10 propyl acetate, and 1:1000 2,4,5-trimethylthiazole were recorded on the same days and so have the same no odor control. Two-sided Wilcoxon rank-sum test with Bonferroni correction (from left to right, *p* = 0.0068, *p* < 0.0001, *p* < 0.0001, *p* < 0.0001, *p* < 0.0001). n = 12-18 recordings, 15 plates each of pyrazine, propyl acetate, and 2,4,5-trimethylthiazole; and 13 no odor plates. Black dot shows data mean. **e**, Change in direction (∆*θ*) executed by animals that start with a large or small angle to the odor (*θ*). Data presented here are the same as in Fig. 1j but additionally include animals that turned away from the odor (negative reorientation angle), demonstrating that amplitude modulation is more notable in animals that have turned in the ‘correct’ direction. n = 18 recording plates. Data show mean ± 95% CI. **f**, Change in direction (∆*θ*) executed by animals that start with a large or small angle to the odor (*θ*). Note that the ‘goal’ for octanol is the opposite of the goal turn for butanone—animals that begin facing toward odor (purple) are best served by executing larger angle turns to turn away from the odor, while animals that begin facing away from the odor (green) are best served by executing small angle turns. We indeed see such a behavior modulation. Two-sided Wilcoxon rank-sum test with Bonferroni correction (*p* = 0.0002, *p* = 0.0002). n = 18 recording plates. Data show mean ± 95% CI. **g**, Fraction of reorientations that turn the animal in the correct dorsal or ventral direction, split by the length of the preceding forward run. Runs are 0–2 s or 2–30 s. Not significant by two-sided Wilcoxon rank-sum test. n = 18 recording plates. Black dots show data mean. **h**, Fraction of reorientations that turn the animal in the correct dorsal or ventral direction among low-angle reorientations, split by reorientation length. Short reorientations are less than 0.5 body lengths. Not significant by two-sided Wilcoxon rank-sum test. n = 18 recording plates. Black dots show data mean. **i**, Fraction of reorientations that move the animal in the correct dorsal or ventral direction, split by the type of postreversal turn executed. Omega turns are >135°, mid-angle turns are 40–135°, and low-angle turns are 0–40°. Not significant by two-sided Wilcoxon rank-sum test with Bonferroni correction. n = 18 recording plates. Black dots show data mean. **j**, Bearing to odor at the end of isolated reorientations, the first reorientation of a pirouette, or the last reorientation of a pirouette for animals in butanone (left, pink) and octanol (right, blue) gradients. Pirouettes are defined as clusters of consecutive reorientations separated by less than 13 s. Two-sided Wilcoxon rank-sum test with Bonferroni correction (from left to right: butanone *p* < 0.0001, *p* = 0.017, *p* < 0.0001; octanol *p* < 0.0001, *p* = 0.0004, *p* < 0.0001). n = 17-18 recording plates per odor. Data are mean ± s.e.m. **k**, Fraction of animals that execute a reorientation in the next 13 s depending on their bearing to the odor at the end of their previous reorientation. Animals that end an individual reorientation in an unfavorable direction (away from butanone or toward octanol) are more likely to reverse again. Two-sided Wilcoxon rank-sum test with Bonferroni correction comparing slopes of the reorientations rate (*p* < 0.0001, *p* < 0.0001). n = 17–18 recording plates. Data are mean ± 95% CI. **l**, Schematic showing the sharp odor gradient. In the sharp gradient, 5–10 animals begin on NGM agar and are surrounded by agar containing the aversive odor octanol (see Methods for more details). By contrast, in the shallow gradient, 20–100 animals begin on minimal agar and are placed equidistant from a distal spot of an odor and a spot of ethanol (which is used to dilute the odor). **m**, Animals can direct reorientations dorsally or ventrally. Which choice is ‘correct’ in a sharp-boundary context depends on the animal’s initial bearing to the aversive octanol boundary. **n**, Example animal encountering octanol during whole-brain calcium imaging, showing that the difference between the baseline and octanol agar is identifiable by eye. The agar boundary is indicated with a black arrow. **o**, Latency to reverse following encounter with the agar boundary in multi-animal recordings. All animals begin on baseline agar and periodically either cross agar with the aversive odor octanol (‘octanol’) or baseline agar (‘no odor’). Each dot is the time in seconds to reverse after each boundary encounter, averaged across all encounters from one plate with 5–10 worms. Two-sided Wilcoxon rank-sum test (*p* < 0.0001). n = 20 recording plates per condition. Data are mean ± s.e.m. **p**, Recording setup for whole-brain calcium imaging. Top, an example animal from NIR imaging, which is used for behavioral data collection. Bottom, an example fluorescent head from spinning disc confocal imaging, which is used to image neuronal activity. To examine aversive olfactory responses, animals begin the recording on baseline agar (NGM) but are surrounded by a sharp octanol gradient. **q**, Fraction of animals that start a reversal in a 10 s interval in whole-brain calcium imaging recordings. ‘Octanol’ specifically looks at whether animals start a reversal in the 10 s following an octanol encounter. ‘Spontaneous’ is from data looking at whether animals reverse in a randomly chosen 10 s interval of spontaneous movement on baseline agar (not octanol). This fraction is calculated by looking at the fraction of animals that reverse in a fixed number of random intervals, which is chosen based on the number of intervals where the animal was on octanol (n = 132). Each dot shows one random sample of data. This process was then repeated 500 times to generate the distribution in black. Statistics compare this distribution to the actual fraction of animals reversing on octanol in a one-sided test. A one-sided test was used as we wanted to test if octanol would increase reversal probability. The octanol value was at the 99th percentile of the dataset (**p < 0.01). **r**, Distribution of signal variation in neuron activity in all recorded datasets compared to previously published GFP controls, recorded under identical image acquisition settings and with the same image processing software for trace extraction^15^. Signal variation is calculated as the s.d. of non-normalized neuron fluorescence divided by the mean of non-normalized fluorescence, as defined in ref. ^15^. This captures the extent to which a given trace shows fluctuations over time. Turning neurons are cells whose activity is associated with head curvature, which are further profiled in Fig. 2. Other neurons are all other recorded GCaMP+ cells in our datasets. The GFP control strain is SWF467, which expresses pan-neuronal GFP and mNeptune. **s**, Sensory neuron activity (ASH, ADL, and AWC) as animals encounter the aversive octanol barrier is shown in blue. Black line shows neuron activity during similar epochs of spontaneous forward movement. ASH is the sensory neuron that senses octanol, AWC senses butanone, and ADL has been shown to have a role in sensing high concentrations of octanol^31,32^. Gray dashed line shows octanol encounter. Two-sided Wilcoxon rank-sum test comparing average activity on octanol and during spontaneous movement postoctanol encounter (*p* = 0.0001), data are mean ± 95% CI. **t**, ASH activity in animals that reverse away from octanol barrier is shown in blue, showing decreasing neuron activity as the animals initiate reversals and successfully leave the octanol. Black line shows ASH activity during spontaneous reverse movement. Black dashed line shows reversal start, and the red shading shows the reversal. Data are mean ± 95% CI. For all panels, significance is noted as: NS (not significant), *P < 0.05, **P < 0.01, ***P < 0.001 and **P < 0.0001. For panels with multiple comparisons, symbols denote Bonferroni-adjusted *P* values.

As a first step toward understanding the neural circuits that control olfactory navigation, we sought to determine the behavioral strategies that *C. elegans* uses to navigate olfactory gradients. To do so, we recorded the locomotion of wild-type (WT) animals as they navigated toward the attractive odor, butanone, or away from the aversive odor, octanol. As a control, we recorded animals on odor-free plates. Previous studies suggested that *C. elegans* navigation relies on two behavioral strategies (Extended Data Fig. 1a)—a ‘biased random walk’ (or klinokinesis) wherein animals heading in an unfavorable direction are more likely to reorient, randomly changing their direction^20^ (Fig. 1a), and ‘weathervaning’ (or klinotaxis), where animals bend their forward movement in a favorable direction^23^ (weathervaning was present in our data; Extended Data Fig. 1b).

To understand navigation behaviors in *C. elegans*, knowledge of the animal’s kinematics is essential. During forward movement, sinusoidal head swings propel body movement forward (Supplementary Video 1). Forward movement can be interrupted by ‘reorientations’, which consist of two sequential motor outputs—a ‘reversal’ (short bout of reverse locomotion), followed by an exaggerated dorsal or ventral head bend that leads to a ‘turn’ as forward movement resumes (Fig. 1a). Reorientations change an animal’s direction of movement and can occur alone or in clusters to form ‘pirouettes’ (Extended Data Fig. 1c).

We first examined whether our data were consistent with a biased random walk by examining how reorientations are influenced by the odor gradient. Consistent with previous results^20^, we found that animals significantly increased their reorientation rate when moving in an unfavorable direction (Fig. 1b,c). We also found that individual reorientations tend to begin 10–20 s after animals have veered in a less favorable direction (Fig. 1d).

We then investigated whether individual reorientations were randomly directed or dependent on the animal’s heading in the olfactory gradient. Furthermore, we examined whether individual reorientations increase or decrease the extent to which animals are moving toward the odor. We found that reorientations may not be simply random—animals improved their bearing through their reorientations, turning toward the attractive odor butanone and turning away from the aversive odor octanol (Fig. 1e,f). Similar effects had been seen in *C. elegans* thermotaxis^28^.

We then examined if this effect was due to animals coupling their heading in the gradient at the onset of reorientations (their direction to the odor, *θ*) to the angle of their reorientation (turn angle, *∆θ*; Fig. 1e). To do so, we performed a shuffle analysis where animals’ initial heading directions were coupled to randomly sampled turn angles from the same video; we then simulated the resulting heading directions. These shuffled data were significantly less likely to improve animals’ bearing in the gradient compared to the real reorientations (Fig. 1e,f). This suggests that individual reorientation turn angles are modulated by the animal’s bearing in the gradient, contradicting the prior assumption of random turning. Past work had hinted at the presence of these directed turns during chemotaxis but lacked the resolution to identify individual reorientation angles^20^.

Animals could improve their bearing by modulating the signs or amplitudes of their reorientations, or both. *C. elegans* lie on their sides when crawling, so their turns are directed either dorsally or ventrally, based on the direction they bend their heads (and thus bodies) during the postreversal turn (Fig. 1a,g). We found that animals navigating to an odor exhibited a small but significant increase in the proportion of reorientations that are in the correct dorsal/ventral (D/V) direction (Fig. 1g,h and Extended Data Fig. 1d). Animals also modulate reorientation amplitude—animals with larger errors in their bearing to the odor gradient (Fig. 1i) executed higher-angle turns than animals with smaller errors at reorientation onset (Fig. 1j and Extended Data Fig. 1e,f). These findings show that animals modulate the signs and amplitudes of their reorientations, executing error-correcting maneuvers that improve their bearing in the odor gradient.

We examined the timescale over which animals collect sensory information to guide their reorientations. The cilia of olfactory neurons are in the tip of the nose, so time-varying changes in nose position in the gradient may be critical. Gradient information could be detected during the dorsal–ventral head swings that propel movement (Fig. 1a) or over slower timescales as the animal displaces its entire body, as has been suggested in past work^25^. We therefore first tested whether longer forward runs improved odor-guided D/V turning during subsequent reorientations. Interestingly, we found no such difference (Extended Data Fig. 1g), suggesting that gradient sensing does not occur exclusively over long timescales during forward runs. Similarly, longer reorientations were no more likely to end in the correct direction than shorter reorientations (Extended Data Fig. 1h). Finally, we found that animals direct reorientations similarly well when executing large or small angle postreversal turns (Extended Data Fig. 1i). Together, these data favor the hypothesis that the sensory gradient is integrated on very short timescales, on the order of just a couple head swings, potentially during both forward and reverse movement (Fig. 1a, time points 1 and 2).

Reorientations can occur as single, ‘isolated’ events or be clustered together to make a ‘pirouette’ that is followed by long periods of forward movement or ‘runs’ (Extended Data Fig. 1c). We found that animals also modulate the direction at which they start their runs based on the gradient direction (Fig. 1k). On average, both isolated reorientations and the last reorientation of a pirouette result in a favorable heading, but the last pirouette reorientation is best aligned with the gradient (Extended Data Fig. 1j). Notably, animals were more likely to reverse again if a reorientation ends in a less favorable direction (Extended Data Fig. 1k). Together, these results suggest that animals both direct their reorientations with respect to the odor gradient (Fig. 1f,h,j and Extended Data Fig. 1d–f) and initiate more reorientations when facing in a bad gradient direction (Fig. 1c,d and Extended Data Fig. 1k).

Finally, we asked whether these behavioral strategies were preserved in environments with different spatial distributions of odors. Inspired by past work^21,29^, we examined *C. elegans* reorientation behaviors when animals abruptly encounter the aversive odor octanol (Extended Data Fig. 1l–n; in contrast to the shallow-gradient experiments above). Of note, the sharp-boundary behavioral recordings followed fewer worms for less time (and animals were only quantified as they moved over the sharp boundary and reoriented) than with the shallow gradients. Because of this smaller sample size, the sharp-boundary data show greater variance than the shallow-gradient data.

Similar to their behavior in shallow gradients, animals were more likely to reverse when moving in an unfavorable direction (Fig. 1l compare to Fig. 1c). They typically reversed after 10–20 s of movement in this unfavorable direction (Extended Data Fig. 1o compare to Fig. 1d). Furthermore, animals performed reorientations that resulted in them pointing away from the odor (Fig. 1m compare to Fig. 1f). Finally, again matching our results above, animals correctly modulated their D/V reorientation direction (Fig. 1n), for example, exhibiting a higher likelihood of ventral turns when the aversive odor was on their dorsal side, similar to a recent description of behavior in other sharp-boundary contexts^29^. These results indicate that *C. elegans* uses related navigation strategies, including directed turning, in both shallow and steep gradients.

### Brain-wide calcium imaging in freely behaving animals surrounded by aversive odors

Our behavioral data revealed that *C. elegans* modulate several aspects of their movement to enable olfactory navigation. We next sought to identify (1) the circuits that integrate information about the sensory gradient, (2) the circuits that encode turn angles, and (3) how these circuits interact. To identify relevant neurons and circuits, we collected brain-wide calcium imaging datasets during spontaneous and odor-guided movement.

We collected data from 32 freely moving animals as they explored the sharp octanol gradients described above. Given the throughput constraints of whole-brain imaging, this design allowed for the collection and pooling of data from many animals experiencing near-identical changes in odor concentration as they moved onto the sharp gradient, something that would have been impossible in shallow gradients.

The recorded animals expressed pan-neuronal NLS-TagRFP and NLS-GCaMP7f (Extended Data Fig. 1p), with NeuroPAL fluorescence barcoding to identify neurons (Fig. 1o,p). Consistent with our behavioral results in the sharp gradient (Fig. 1l), the recorded animals showed elevated reorientation rates after their heads crossed onto the octanol agar (Extended Data Fig. 1q). These data were recorded and processed using previously described custom-built tracking software, image analysis software and neuron identification methods^15,18,30^. Our previous work showed negligible motion artifacts^15,18,30^, which we further confirmed here (Extended Data Fig. 1r).

From the 32 recorded animals, we obtained activity data for an average of 102 identified neurons per animal. We first confirmed that our data captured the dynamics of neurons whose activity changes during navigation-relevant behaviors such as head bending (examples in Fig. 1o,p compare to Fig. 1a). We also confirmed that the octanol sensory neuron ASH increased activity upon octanol encounter and diminished as animals reversed away from octanol (Extended Data Fig. 1s,t). We observed no change in the activity of other sensory neurons such as AWC, which senses butanone^31^, or ADL, which senses higher concentrations of octanol than used here^32^ (Extended Data Fig. 1s). We then sought to identify neural signatures in our brain-wide recordings that correspond to the navigation strategies seen in our behavioral data.

### Neurons in the head-steering circuit encode sensory information and turning directions

Our behavioral data suggested that animals regulate the timing and angles of reorientation based on sensory cues. We next examined the precise neural dynamics associated with these behaviors. We focused on the neurons of the head-steering circuit for several reasons. First, these neurons have dorsal and ventral counterparts (for example, SMBD and SMBV) that differentially innervate dorsal and ventral head muscles, thereby enabling them to direct D/V head bending. Exaggerated dorsal or ventral head bends are critical to direct postreversal turn directions. Second, behavioral studies have found that the neurons in this circuit are required for maintaining normal head curvature^15,33–39^ and many are required for postreversal turns^33,34,40^. Their causal roles in these spontaneous behaviors, which are heavily used during navigation, make them excellent candidates for odor-guided behaviors. Third, head-steering neurons’ activities oscillate with head bending, although these dynamics differ depending on whether the animal is moving forward or reversing^15,34^. Neurons with such dynamics are candidates for representing the sensory gradient, as they can combine incoming sensory inputs with head-bending information. Thus, we hypothesized that the neurons of the head-steering circuit may show changes in dynamics based on turn direction, the sensory gradient or both.

**Fig. 2:**
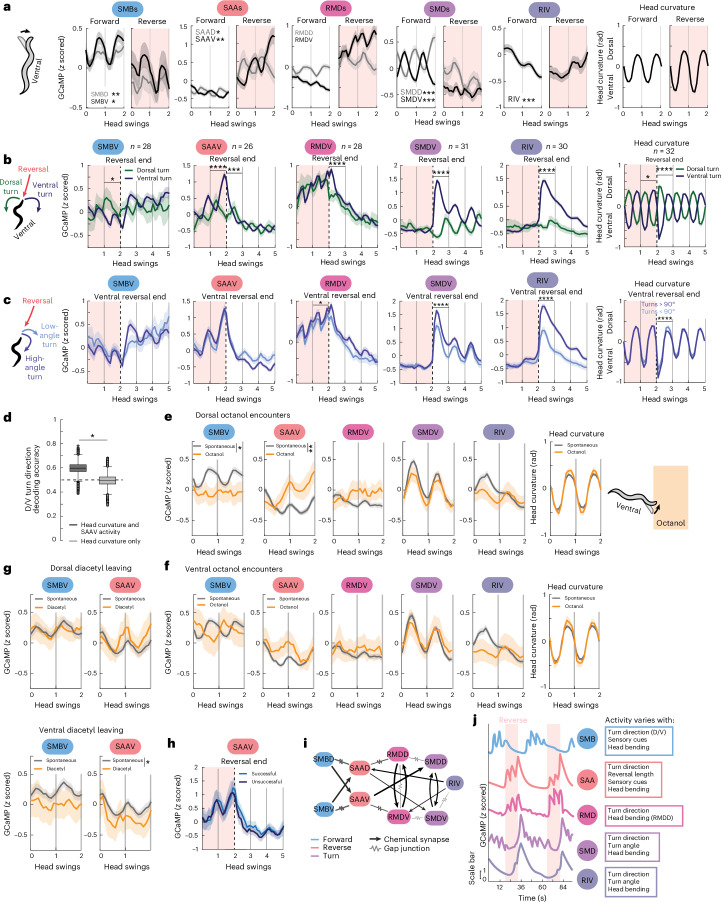
The neurons of the head-steering circuit are sequentially activated during reorientations, encoding the signs and amplitudes of turns. **a**, Head-curvature-associated neuron activity during forward or reverse movement. Neural activity in this plot is aligned to head swings, as head-swing frequency can vary across reorientations and animals. More details of alignment can be found in Methods. Data are mean ± 95% CI. From left to right, fraction of datasets where neuron activity significantly encoded head curvature—SMBV, 33%; SMBD, 46%; SAAV, 48%; SAAD, 38%; RMDV, 17%; RMDD, 10%; SMDV, 77%; SMDD, 66% and RIV, 67%. The head-curvature encoding was calculated as defined in ref. ^15^. An asterisk indicates neuron significantly encodes head curvature in more than 20% datasets; double asterisks indicate neuron significantly encodes head curvature of >40% datasets and triple asterisks indicate neuron significantly encodes head curvature of >60% datasets. **b**, Neuron activity throughout reorientations, shown as event-triggered averages aligned to reversal endings, split by turn direction. Activity is aligned to head curvature as in **a** and Methods. *n* = 99–150 dorsal turn and 462–554 ventral turn reversals (*n* values on the plot show the number of recordings with data for that neuron). Two-sided Wilcoxon rank-sum test with Bonferroni correction, comparing activity one head swing (~5 s) before or after the reversal end (from left to right, *P* = 0.0013, *P* < 0.0001, *P* < 0.0001, *P* < 0.0001, *P* < 0.0001, *P* < 0.0001, *P* = 0.0011, *P* < 0.0001). Data are mean ± 95% CI. **c**, Neuron activity during high (>90°) and low (<90°) angle ventral turns, with data displayed similarly to **b**. *n* = 392–472 reversals in total. Two-sided Wilcoxon rank-sum test with Bonferroni correction comparing activity one head swing (~5 s) before or after the reversal end (from left to right, *P* = 0.0025, *P* < 0.0001, *P* < 0.0001, *P* < 0.0001). Data are mean ± 95% CI. **d**, Test accuracy of RNNs trained to predict postreversal turn direction based on neural activity and/or behavior during the reversal. (For additional details, see Extended Data Fig. 2d and Methods.) *P* = 0.0466, one-sided binomial test used to generate an empirical *P* value that SAAV activity improved decoding accuracy, based on bootstrapping (Methods). Box plot shows median and IQR, error bars show Q1 and Q4, dots show points outside this range. **e**, Neuron activity during head swings where the animal was moving forward onto the octanol gradient compared to similar spontaneous head swings. This panel considers dorsal odor encounters, which occur when the odor is on the dorsal side of the head when the animal makes its first head swing on odor. Activity is aligned with head swings, as in **a**. Statistics compare average activity during dorsal octanol approach versus spontaneous. Two-sided Wilcoxon rank-sum test with Bonferroni correction (SMBV, *P* = 0.0046; SAAV, *P* = 0.0005), data are mean ± 95% CI. **f**, Same as **e**, but for events when the animal approaches octanol on its ventral side. Data are mean ± 95% CI. **g**, Same as **e** and **f**, but comparing activity when the animal exits the attractive odor diacetyl. Two-sided Wilcoxon rank-sum test with Bonferroni correction (*P* = 0.0103), data are mean ± 95% CI. **h**, SAAV activity (**b**) in reorientations followed by ventral turns, split by whether the next reorientation navigated off of octanol (‘successful’) or resulted in the animal remaining on octanol (‘unsuccessful’). *n* = 39 successful reversals, 59 unsuccessful reversals. Activity is not significantly different in the first head swing before or after reversal end using the Wilcoxon rank-sum test with Bonferroni correction. **i**, Connectivity among the neurons in the head-steering circuit (data from refs. ^11,12^). **j**, Mock traces of head-steering neurons’ activities, annotated with the behavioral and sensory features that influence each neuron’s activity. IQR, interquartile range. For all panels except (**a**), significance is noted as: NS (not significant), *P < 0.05, **P < 0.01, ***P < 0.001 and **P < 0.0001. For panels with multiple comparisons, symbols denote Bonferroni-adjusted *P* values.

We first examined how head-steering circuit neurons vary their activity with head swings during spontaneous forward and reverse movement. These stereotyped dorsal–ventral head swings propel the body forward or backward, generating a wave of motion along the worm’s body (Fig. 1a). They may also underlie odor gradient sampling. Consistent with prior work, SMD neurons oscillate with head bending, with especially high activity during forward movement (Fig. 2a,b; note that neural activity here is aligned to head swings; figure legend and Methods). Our data also revealed several new aspects of neural activity. SAA neurons oscillate with head bending and dramatically scale up their oscillations throughout reversals (Fig. 2a). RMDD (but not RMDV) neurons invert the phase of their oscillations relative to head bending in forward versus reverse movement (Fig. 2a). SMB neurons oscillate with head bending and reduce their activity during reversals (Fig. 2a). These results suggest that head-steering neurons’ relationship between activity and head-bending remaps based on the movement direction.

**Extended Data Fig. 2:**
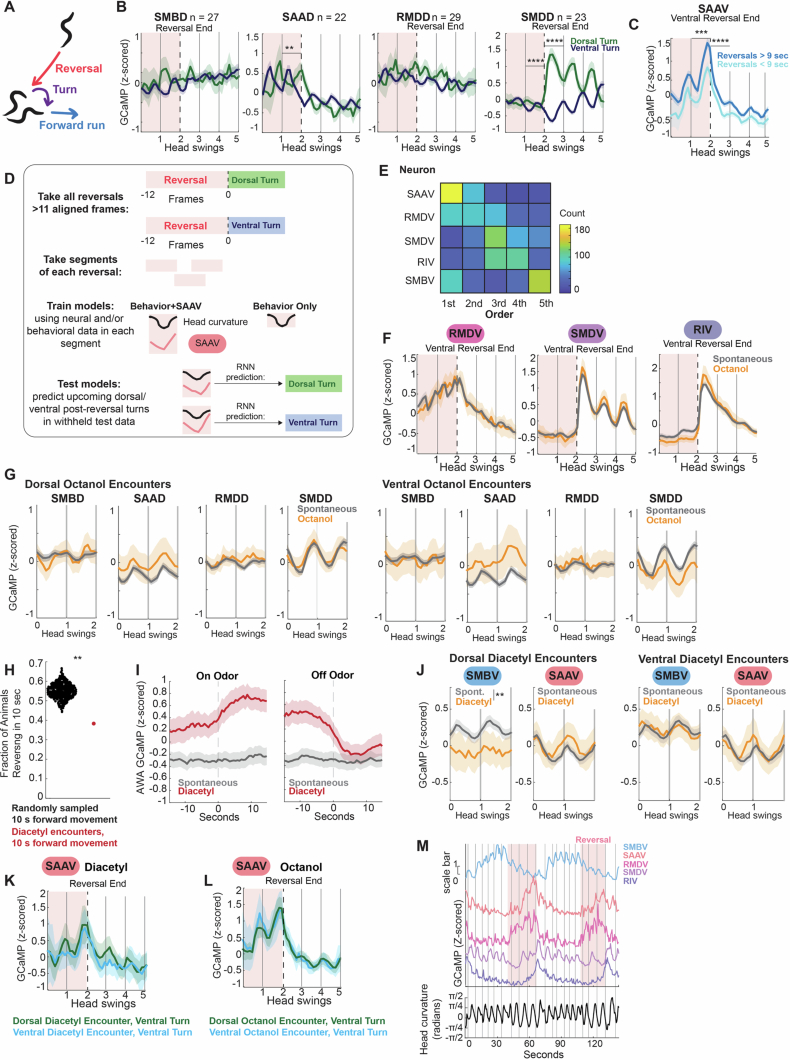
Further analysis of neural circuit dynamics in turning circuit. **a**, Reorientations are composed of a period of backwards velocity (a reversal), shown in red; then a high-angle turn as the animal moves forward, shown in purple; then the forward run, shown in blue. **b**, Dorsal counterparts of the neurons shown in Fig. 2b, showing neuron activity across reorientations. Red shading shows reversal; black dashed line is at reversal end. Z-scored activity is aligned to head curvature, data are separated into reversals with dorsal vs ventral postreversal turns. Further alignment description found in Fig. 2b and Methods. n = 115–140 dorsal turn and 415–524 ventral turn reversals. Two-sided Wilcoxon rank-sum test with Bonferroni correction, comparing activity 5 s (one head swing) before or after the reversal end (from left to right, *p* = 0.001, *p* < 0.0001, *p* < 0.0001). Data are mean ± 95% CI. **c**, Average SAAV activity aligned to reversal ends during long and short reorientations with ventral postreversal turns. Red shading shows reversal; black dashed line is at reversal end. Z-scored activity is aligned to head curvature. Data are split by reversal length. SAAV activity is higher in longer reversals (>9 s), reflecting that it ramped to a higher activity level during these longer reversals. n = 462 reversals. Two-sided Wilcoxon rank-sum test (both *p* < 0.0001), comparing 5 s (one head swing) before or after the reversal end. Data are mean ± 95% CI. **d**, Approach used for decoding of upcoming turn direction. Aligned head curvature and SAAV activity (as in Fig. 2b) were taken from all reversals 12 frames or longer (~1.5 head swings). Time segments of activity and behavior of length 4 (that is, 4 frames, which is 2.4 s) were then extracted from these reversals. These segments were then used to validate, train, and test recurrent neural networks (RNNs) with fivefold cross-validation (more information can be found in Methods) to predict the upcoming postreversal turn direction. We then compared the decoding accuracy of an RNN trained on behavior and SAAV activity to that of an RNN trained on behavior alone. See Methods for additional details. **e**, Order in which each neuron reaches its peak activity across all recordings with these five neuron classes captured (SAAV, RMDV, SMDV, RIV, and SMBV). To determine the activity order, the time at which each neuron’s activity is highest during the transition between reversal to turn forward was quantified (here we examined all neuron activity from 1.8 s before the reversal end to 7.2 s after the reversal end). Based on these times, the order in which the neurons were most active in that reorientation was assigned (first, second, etc.). n = 190 reorientations. **f**, Neuron activity as animals reverse away from octanol barrier is shown in orange, specifically showing reversals followed by a ventral turn that start on octanol and end on NGM agar (and therefore navigate the animals away from the odor). The gray line shows neuron activity during spontaneous reverse movement followed by a ventral turn. Black dashed line shows reversal end; reversals are shaded in red, as in Fig. 2b. Data are mean ± 95% CI. RMDV n = 36 octanol reversals, 488 spontaneous. SMDV n = 49 octanol reversals, 555 spontaneous. RIV n = 45 octanol reversals, 550 spontaneous. **g**, Neuron activity during head swings where the animal was moving forward onto the octanol gradient, as in Fig. 2e,f, showing the dorsal counterpart neuron activity. Neural activity during these head swings was compared to activity during similar spontaneous head swings on baseline agar. Dorsal and ventral octanol encounters are defined by the direction of the first head swing that the animal makes when they encounter the octanol boundary (see diagram at **e**). Neuron activity is aligned to the head swings, as described in Fig. 2a. Data are mean ± 95% CI. None of the comparisons is significant using the Wilcoxon rank-sum test with Bonferroni correction. **h**, Fraction of animals that start a reversal in a 10 s interval. ‘Diacetyl’ examines whether animals start a reversal in the 10 s following a diacetyl encounter. ‘Spontaneous’ examines if animals reverse in a randomly chosen 10 s interval of spontaneous movement on baseline agar (not diacetyl). This fraction is calculated by looking at the fraction of animals that reverse in a fixed number of random intervals, which is chosen based on the number of intervals where the animal was on diacetyl. Each dot shows one random sample of data. This process was then repeated 500 times to generate the distribution in black. Statistics compare this distribution to the actual fraction of animals reversing on octanol in a one-tailed test (**p < 0.01). **i**, AWA activity upon diacetyl encounter (left) and as the animal leaves diacetyl (right). To control for motion-responsive effects, the ‘spontaneous’ data are from epochs of similar forward versus reverse movement off odor. For each individual odor encounter or exit event, the two most similar epochs of movement where the animal was consistently off odor were chosen across all recorded videos. These two were then added to the ‘spontaneous’ distribution. AWA activity is high before diacetyl encounter because animals frequently have recently been on diacetyl before they return to it. Data are mean ± 95% CI. **j**, Neuron activity during head swings where the animal was moving forward, entering diacetyl. Neural activity during these head swings was compared to activity during similar spontaneous head swings on baseline agar. As in Fig. 2e–g, these panels are split by the direction of approach at the moment of odor leaving. Neuron activity is aligned to the head swings, as described in Fig. 2a. Statistics compare average neuron activity during ventral/dorsal diacetyl exit versus spontaneous movement. Two-sided Wilcoxon rank-sum test with Bonferroni correction (*p* = 0.0006), data are mean ± 95% CI. **k**, SAAV activity during the first reversal after the animal encounters diacetyl, split by the direction the animal encounters diacetyl (direction of approach as in Fig. 2e,f, activity presented as in Fig. 2b). Data are mean ± 95% CI. n = 33–36 reversals. Activity is not significantly different in the first head swing before or after reversal end using two-sided Wilcoxon rank-sum test with Bonferroni correction. **l**, SAAV activity during the first reversal after the animal encounters octanol, split by the direction the animal encounters, as in **k**. Data are mean ± 95% CI. n = 25 ventral encounter reversals, 50 dorsal encounter reversals. Activity is not significantly different in the first head swing before or after reversal end using two-sided Wilcoxon rank-sum test with Bonferroni correction. **m**, Example dataset with joint activity recordings of single SAAV, RMDV, SMDV, RIV, and SMBV neurons over two reorientations. Red shading shows reversals, gray lines show when head curvature crosses from dorsal to ventral (positive to negative). Head curvature for the same animal is quantified at the bottom. For all panels, significance is noted as: NS (not significant), *P < 0.05, **P < 0.01, ***P < 0.001 and **P < 0.0001. For panels with multiple comparisons, symbols denote Bonferroni-adjusted *P* values.

We then focused on identifying neurons whose activity changes with turn direction or amplitude. Our goal was to identify candidate cells that may direct these behaviors during navigation. We first considered whether neuronal activity in the head-steering circuit differs during dorsal versus ventral turns, as has been shown for SMD^34,41^. Here we discuss data for the ventral (V) neuron class for each cell type, but related trends were also seen for these cells’ dorsal (D) counterparts (Extended Data Fig. 2b). SMDV, RMDV and RIV were active during turns and displayed higher activity during ventral turns compared to dorsal (Fig. 2b). SMDV and RIV also displayed increased activity during higher-angle ventral turns compared to lower-angle ventral turns (Fig. 2c). This suggests that RMDV, SMDV and RIV are active during turns and encode the turn properties, or ‘kinematics’. By contrast, SAAV activity was higher during reversals that ended in ventral (compared to dorsal) turns, displaying this activity difference before the turns were actually executed (Fig. 2b and Extended Data Fig. 2c). This suggests that SAAV may act to bias or predict the upcoming turn direction.

Our SAAV results suggested the possibility that neural activity might be able to predict upcoming turning behavior. We then sought to determine whether SAAV activity was predictive of future behavior or, alternatively, whether head bending during the reversal predicted the upcoming turn direction. To test this, we attempted to predict upcoming turn direction from head curvature and/or SAAV activity during the reversal using a recurrent neural network (RNN) with fivefold cross-validation (Extended Data Fig. 2d; Methods). The RNN trained on both behavior and SAAV activity was significantly better at decoding upcoming turn direction than that trained on behavior alone (Fig. 2d). This suggests that SAAV activity does carry information relevant to future turning behavior.

We then examined whether the head-steering circuit neurons respond to sensory input. The recorded animals in our calcium imaging data occasionally encountered the aversive odor octanol, and our sharp-boundary paradigm ensured that the odor encounter was relatively standardized. We asked whether the oscillations of these neurons were altered when the animal encountered octanol (Fig. 2e,f). As we were interested in which neurons control directed turns, we specifically examined if activity differed based on whether the animals encountered the sharp-octanol gradient with the odor primarily on their dorsal side (Fig. 2e) or their ventral side (Fig. 2f). If a cell is able to encode information about the sensory gradient, it should respond differently to these approach directions. By contrast, if a cell responds similarly to both directions, it may generically respond to the aversive odor cue. Indeed, we observed sensory-dependent and direction-dependent changes in SMBV and SAAV activity. The neurons that encoded kinematics, SMDV, RMDV and RIV, did not exhibit responses to the octanol (Fig. 2e,f), but instead continued to faithfully encode the resulting odor-guided turn direction (Extended Data Fig. 2f).

The response in SAAV was of particular interest. When animals approached the aversive odor octanol on the dorsal side, SAAV activity increased significantly (Fig. 2e). By contrast, when animals approached octanol with the odor on their ventral side, SAAV activity decreased (Fig. 2f). As SAAV activity is higher during reversals preceding ventral turns (Fig. 2b), these results make intuitive sense—when animals approach octanol dorsally, a ventral postreversal turn will move them in the correct direction, away from octanol. We also observed that SAAD activity was highest as the animals approached the octanol ventrally (Extended Data Fig. 2g; although this effect was only a trend, as it did not reach significance).

We then examined whether these results generalized to other odors and valences. We therefore designed a sharp-odor paradigm in which animals periodically encountered the attractive odor diacetyl. Animals reduced their reorientation rate upon encountering diacetyl (Extended Data Fig. 2h), a typical response of *C. elegans* to an attractive odor. Additionally, the diacetyl sensory neuron, AWA, significantly increased activity upon diacetyl encounter and decreased activity after exiting diacetyl (Extended Data Fig. 2i). We found that SAAV activity changed upon diacetyl exit similarly to our octanol encounter results—its activity was suppressed when animals left diacetyl ventrally, but trended upwards when it left diacetyl dorsally (Fig. 2g). This again would be predicted to promote advantageous turn directions back toward the diacetyl. This suggests that this neural response generalizes across odors. In contrast, SAAV activity did not change when animals moved onto an attractive odor (Extended Data Fig. 2j), suggesting that SAAV encodes sensory gradient information only under conditions likely to result in an upcoming reversal.

The ramping of SAAV during reversals could suggest that SAAV activity is elevated by reverse locomotion, perhaps to bias the upcoming turn direction. However, an alternative explanation could be that SAAV encodes an error signal with respect to the odor gradient. To test this, we first examined whether SAAV activity differs when reorientations turn the animal in the correct versus incorrect direction after octanol encounter. No differences were observed (Extended Data Fig. 2k,l). We also examined whether SAAV activity changed when animals executed a reorientation that successfully navigated animals off of octanol, compared to reorientations that resulted in the animals staying on octanol. We observed no change in SAAV activity based on reorientation success (Fig. 2h). This suggests that, while SAAV shows odor-modulated activity, its activity is not related to reversal outcomes. We currently favor the model that SAAV activity encodes sensory gradient information when animals are likely to make an upcoming reorientation.

Taken together, these observations suggest that neural activity in the head-steering circuit evolves in a stereotyped, sequential order that depends on the properties of the turn (Fig. 2i,j). (We define this reliable, ordered progression of neuron activity as a ‘neural sequence’.) During forward movement, SMB and SAA activity oscillations are influenced by the odor gradient. As reversals begin, SAA and RMD oscillations increase, with SAA reporting the upcoming turn direction. When the turn begins, SAA activity falls, and RMD, SMD and RIV encode the turn angle. After the turn, SMB oscillations begin again. This sequence can be observed in aggregate data (Fig. 2a–c and Extended Data Fig. 2b,e) and single animals (Extended Data Fig. 2m). Because these neurons vary their activity based on navigation-related factors, these results strongly suggested that these neurons may causally contribute to navigation behavior.

### Head-steering circuit neurons causally affect spontaneous and odor-guided reorientations

**Fig. 3:**
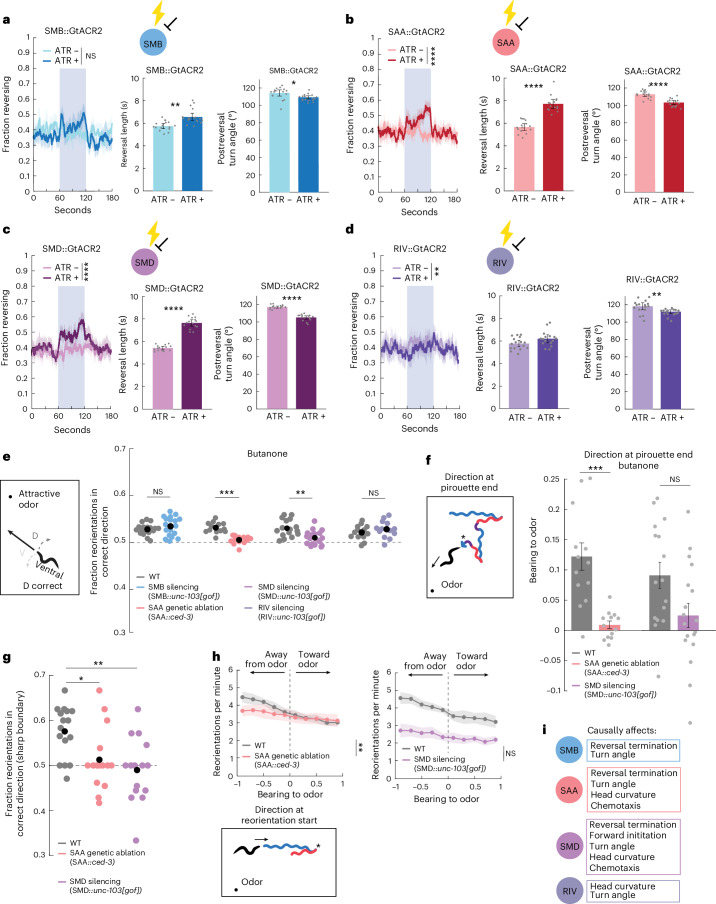
Head-steering circuit neurons causally affect spontaneous and odor-guided reorientations. **a**–**d**, Behavior during optogenetic inhibition of the SMBs (**a**), SAAs (**b**), SMDs (**c**) or RIVs (**d**). Cell-specific promoters were used to express the optogenetic silencing channel GtACR2 in each cell class. SMB is *flp-12(short fragment)::GtACR2-sl2-GFP*; SAA is intersection of *lad-2::cre* *+* *unc-42::inv(GtACR2-sl2-GFP)*; SMD is intersection of *lad-2::cre* *+* *fkh-10::inv(GtACR2-sl2-GFP)*; and RIV is intersection of *lad-2::cre* *+* *sri-5::inv(GtACR2-sl2-GFP)*. Promoter specificity was validated by GFP coexpression. From left to right, the panels for each neuron show—fraction of animals reversing (shading shows blue light optogenetic inhibition); reversal length; and postreversal turn angle during optogenetic stimulus. SMB, *n* = 18 plates ± ATR; SAA, *n* = 15 plates ± ATR; SMD, *n* = 15 +ATR, 14 −ATR; RIV, *n* = 16 plates ± ATR. Recorded plates of 20–100 animals, 6 optogenetic stimulations per recording (only the first stimulation is shown for the fraction reversing plots, although all are combined for statistics). Two-sided Wilcoxon rank-sum test with Bonferroni correction, comparing with and without ATR. (From left to right—SMB, *P* = 0.0795, *P* = 0.0002, *P* = 0.0055; SAA, *P* < 0.0001, *P* < 0.0001, *P* < 0.0001; SMD, *P* < 0.0001, *P* < 0.0001, *P* < 0.0001; RIV, *P* = 0.0016, *P* = 0.1, *P* = 0.0031.) For all plots, data are mean ± 95% CI. **e**, Fraction reorientations in the correct D/V direction during butanone chemotaxis for animals lacking a specific neuron compared to WT animals. (From left to right—SMB silenced, SAA genetic ablation, SMD silenced, and RIV silenced.) SAA genetic ablation is intersection promoter consisting of *lad-2::ced-3(p15) + unc-45::ced-3(p17)*. The two *ced-3* subunits combine to form a functional caspase, leading to the cell death of SAA only^15,36^. SMB silencing is *flp-12(short fragment)::unc-103[gof]*, SMD silencing is intersectional promoter consisting of *lad-2::cre* *+* *fkh-10::inv(unc-103[gof])* and RIV silencing is intersectional promoter with *lad-2::cre* *+* *sri-5::inv(unc-103[gof])*. Cell-specific strains were run on separate days, so each has its own WT control. *n* = 13–18 recording plates. Two-sided Wilcoxon rank-sum test with Bonferroni correction (from left to right, *P* = 0.319, *P* < 0.0001, *P* = 0.0023, *P* = 0.383). Black dots show data mean. **f**, Bearing to odor at pirouette end during butanone chemotaxis for SAA genetically ablated (left) or SMD silenced (right) versus WT animals. SAA, *n* = 13 plates each of WT and genetic ablation, *P* = 0.0002. SMD, *n* = 16 WT and 18 SMD silencing plates, *P* = 0.0261. Two-sided Wilcoxon rank-sum test with Bonferroni correction. Data show mean ± s.e.m. **g**, Fraction reorientations in the correct D/V direction during a sharp-octanol boundary encounter (Fig. 1n) for SAA genetically ablated, SMD silenced and WT animals. Recordings were all run on the same day. *n* = 15–16 recordings. Two-sided Wilcoxon rank-sum test with Bonferroni correction (SAA, *P* = 0.0166; SMD, *P* = 0.0028). Black dots show data mean. **h**, Reorientation rate compared to bearing to odor during butanone chemotaxis for SAA genetically ablated (left) or SMD silenced (right) versus WT animals. *n* = 13–18 recording plates. Two-sided Wilcoxon rank-sum test with Bonferroni correction comparing slopes of the rate of reorientation initiation (SAA, *P* = 0.0006; SMD, *P* = 0.051). Data show mean ± 95% CI. **i**, Summary of each head-steering circuit cell’s functional role. ATR, all-*trans*-retinal. For all panels, significance is noted as: NS (not significant), *P < 0.05, **P < 0.01, ***P < 0.001 and **P < 0.0001. For panels with multiple comparisons, symbols denote Bonferroni-adjusted *P* values.

**Extended Data Fig. 3:**
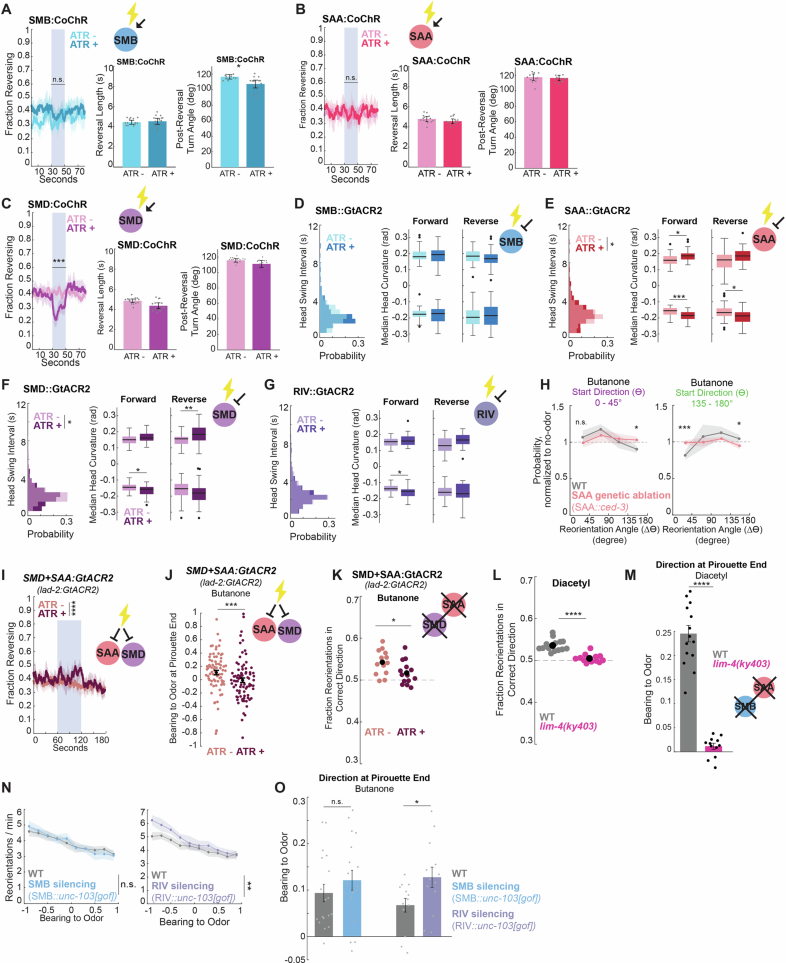
Additional neural perturbations during navigation behavior. **a**–**c**, Behavioral effects of optogenetically activating the SMBs, SAAs, or SMDs. Cell-specific promoters were used to express the excitatory optogenetic CoChR channel in each cell class, as described in Fig. 3a–d. From left to right for each neuron: fraction of animals reversing across time, with the shaded bar showing optogenetic activation; reversal length; postreversal turn angle. SMB n = 13–14 plates, SAA n = 13 plates per condition, SMD n = 14–15 plates, RIV n = 13–16 plates. For all strains, there are 7 optogenetic stimulations per recording. Two-sided Wilcoxon rank-sum test with Bonferroni correction, comparing with and without ATR (SMB *p* = 0.002, SMD p < 0.0001). For all plots, data are mean ± 95% CI. **d**–**g**, Behavioral effects of optogenetically silencing the SMBs, SAAs, SMDs, or RIV. Cell-specific promoters were used to express the excitatory optogenetic Gt2 channel in each cell class. SMB is *flp-12(short fragment)::GtACR2-sl2-GFP*; SAA is *lad-2::cre* *+* *unc-42::inv(GtACR2-sl2-GFP);* SMD is *lad-2::cre* *+* *fkh-10::inv(GtACR2-sl2-GFP)*; and RIV is *lad-2::cre* *+* *sri-5::inv(GtACR2-sl2-GFP)*. For each neuron, the left shows time interval between successive head swings for all head swings during optogenetic stimulation, comparing with or without ATR. The right is the median head curvature during each optogenetic stimulation in radians during forward or reverse movement. Negative values are dorsal head swings and positive values are ventral head swings. SMB n = 10 animals per condition; SAA n = 9 +ATR, 13 −ATR animals; SMD n = 14 +ATR, 13 −ATR animals; RIV n = 8 +ATR, 9 −ATR. In each recording (that is, animal), there were four 1 min optogenetic stimulations. Data recorded using the single worm trackers (Methods). Two-sided Wilcoxon rank-sum test with Bonferroni correction (from left to right, SAA *p* = 0.0076, *p* = 0.0024, *p* < 0.0001, *p* = 0.0057; SMD *p* = 0.0093, *p* = 0.0046, *p* = 0.0005; RIV *p* = 0.0078). For box plots, data show median, IQR, and range. **h**, Change in direction (∆*θ*) executed by animals that start with a small (left, purple) or large (right, green) angle direction to the odor (*θ*), normalized to no odor controls of the same genotype. Note that SAA-ablated animals are different from WT on both small and large reorientations, indicating a lack of modulation of turn amplitude based on gradient information. Two-sided Wilcoxon rank-sum test with Bonferroni correction (from left to right, *p* = 0.124, *p* = 0.0025, *p* = 0.0002, *p* = 0.0029). Data show mean ± 95% CI. **i**, Behavioral effects of optogenetically inhibiting both the SMDs and SAAs (as well as SDQ, PLN, ALN, expression based on ref. ^36^). Animals express *lad-2::GtACR2*. Graph shows fraction of animals reversing across time. n = 13–14 recording plates, 6 optogenetic stimulations per recording. Two-sided Wilcoxon rank-sum test (p < 0.0001). Data are mean ± 95% CI. **j**, Bearing at the ends of pirouettes during butanone chemotaxis in *lad-2::GtACR2* animals. Only pirouettes that end during the optogenetic inhibition were included in this analysis. Two-sided Wilcoxon rank-sum test comparing mean bearing to odor per stimulation with and without ATR (*p* = 0.0006). n = 13 −ATR and 14 +ATR recordings each with 6 stimulations. Data are mean ± s.e.m. **k**, Fraction animals making the correct dorsal versus ventral turn in a butanone gradient in *lad-2::GtACR2* animals. Only reversals that end during the optogenetic inhibition were included in this analysis. Each dot is one plate with 20–100 animals. Two-sided Wilcoxon rank-sum test (*p* = 0.0272). n = 13–14 recordings. Each dot shows average value from all reversals that end during any of the six stimulations in a recording. **l**, Fraction reorientations in the correct dorsal/ventral direction during diacetyl chemotaxis for *lim-4* mutant vs wild type animals. *lim-4(ky403)* animals are cell fate mutants that result in a cell fate change for the SMB neurons, among other cells^37^, and morphological deficits in the SAA neurons^42^. In these animals, an aversive sensory neuron, AWB, takes on the cell fate of the butanone-sensing sensory neuron AWC^42^. Therefore, we used the odor diacetyl, which is sensed by the sensory neuron AWA^31^, to test these animals’ behavior, as past work has shown that *lim-4* mutants can respond to diacetyl^42^. Wild-type data here are also shown in Extended Data Fig. 1d. n = 12–14 recording plates. Two-sided Wilcoxon rank-sum test (*p* < 0.0001). Black dots show data mean. **m**, Bearing at the ends of pirouettes during diacetyl chemotaxis in *lim-4(ky403)* animals. n = 14 wild type and 12 *lim-4(ky403)* recording plates. Two-sided Wilcoxon rank-sum test (*p* < 0.0001). Data are mean ± s.e.m. **n**, Reorientation rate compared to bearing to odor during butanone chemotaxis for SMB silenced (left) or RIV silenced (right) vs wild type animals. RIV-silenced animals were more likely to increase their reorientation rate when facing away from the odor. Two-sided Wilcoxon rank-sum test with Bonferroni correction comparing slopes of the rate of reorientation initiation. SMB n = 18 plates per genotype, *p* = 0.09; RIV n = 13 plates per condition, *p* = 0.001. Data show mean ± 95% CI. **o**, Bearing to odor at pirouette end during butanone chemotaxis for SMB silenced (left) or RIV silenced (right) vs wild type animals. Two-sided Wilcoxon rank-sum test with Bonferroni correction. SMB n = 18 plates per genotype, *p* = 0.304; RIV n = 13 plates per condition, *p* = 0.021. Data show mean ± s.e.m. For all panels, significance is noted as: NS (not significant), *P < 0.05, **P < 0.01, ***P < 0.001 and **P < 0.0001. For panels with multiple comparisons, symbols denote Bonferroni-adjusted *P* values.

Our calcium imaging data indicated that many of the cells of the head-steering circuit change activity during behaviors relevant for navigation, like head swings, reorientations and odor-guided turning (Fig. 2j). We then tested if these cells have direct control over these behaviors. We first examined spontaneous behavior. We assessed how optogenetically silencing these neurons affected head-swing frequency and amplitude, reversal speed, reversal length and postreversal turn angle. Optogenetically silencing the SMBs, SAAs, SMDs or RIVs resulted in changes to at least one of these behavioral features (Fig. 3a–d and Extended Data Fig. 3a–g). Several classes contribute to the termination of reversals, consistent with past work on the SAAs and SMDs^34,39^, and all contribute to postreversal turn amplitudes (Fig. 3a–d). These neurons have slightly different, and possibly redundant, roles; the largest effect sizes were seen when silencing SAA or SMD.

We then examined navigation behavior. Animals lacking either a functional SAA or SMD executed significantly fewer D/V-directed turns (Fig. 3e). Turn direction selection was unaffected when SMB or RIV was silenced (Fig. 3e). SAA-ablated animals also showed diminished modulation of turn amplitude (Extended Data Fig. 3h); began forward runs in apparently random directions (Fig. 3f); did not regulate their D/V reorientation direction in a sharp-boundary arena (Fig. 3g); and were less likely to regulate reorientation starts based on odor cues (Fig. 3h). Joint optogenetic silencing of SMD and SAA yielded similar results (Extended Data Fig. 3i–k). Additionally, *lim-4* mutants, which have morphological deficits in SAA^42^ and cell fate deficits in SMB^37^, among others, began forward movement less well aligned to the odor gradient than WT animals (Extended Data Fig. 3l,m). Overall, these results establish a causal role for SAA and SMD neurons in controlling odor-guided turning.

The roles of SMB and RIV during navigation remain less clear. Silencing the SMBs alone did not affect quantified navigation behaviors (Extended Data Fig. 3n,o), although SMBV exhibits odor-modulated activity in response to both attractive and aversive odors (Fig. 2e–g and Extended Data Fig. 2j). This suggests that the signal from SMB may be sent in parallel to other sensory signals.

Together with the above results (Fig. 2j), these data suggest that each cell in the head-steering circuit is important for ending reversals and determining turn angles (Fig. 3i). In particular, these results highlight a functional requirement for two key neurons in the neural sequence, SAA and SMD, and suggest modulatory functions for the other neurons.

### Forward-active neurons can control spontaneous and odor-guided reorientation timing

**Fig. 4:**
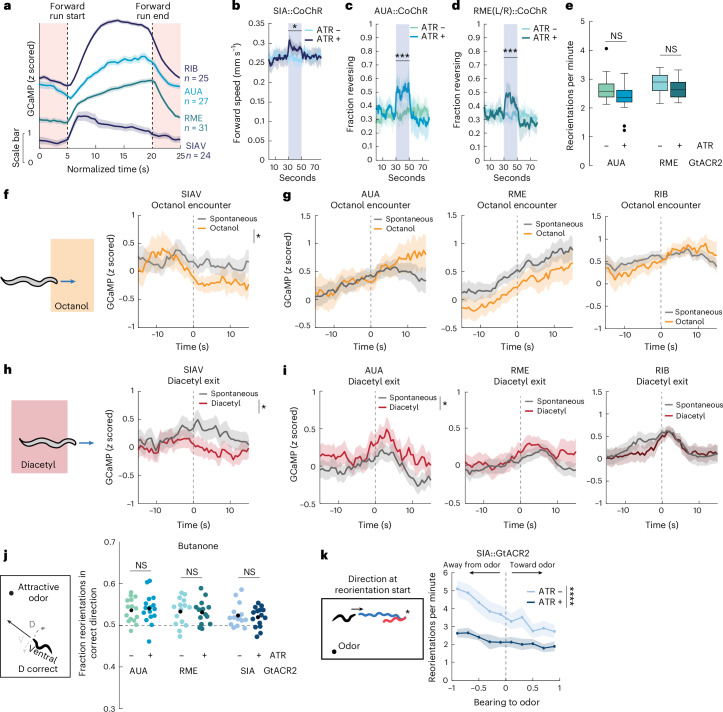
Forward-active neurons exhibit varied dynamics and behavioral roles. **a**, Neuron activity across entire forward runs. Data are from runs that were 10–20 s (~40% of runs); *z*-scored neuron activity during the run was linearly compressed or expanded to align with a standard 15 s run. Red shading shows reversals. *n* = 54–272 runs (*n* values on the plot show the number of recordings with data for that neuron). Data are mean ± 95% CI. **b**, Change in forward speed during optogenetic activation of SIA neurons. SIA promoter is the intersection of *ceh-17::cre* + *pdf-1::inv(CoChR-sl2-GFP)*. *n* = 14 −ATR and 15 +ATR recordings, 7 optogenetic stimulations per recording (only the first stimulation is shown for the fraction reversing plots, although all are combined for statistics). Two-sided Wilcoxon rank-sum test, comparing average speed ± ATR (*P* = 0.0176). Data are mean ± 95% CI. **c**,**d**, Fraction of animals reversing over time during single neuron optogenetic activation of AUA (**c**) or RME (**d**). AUA promoter is intersection of *ceh-6::cre* *+* *flp-8::inv(CoChR-sl2-GFP)*; RME promoter is intersection of *vap-1::cre* *+* *unc-25::inv(CoChR-sl2-GFP)*. Promoter specificity was validated by GFP coexpression. AUA, *n* = 9 plates per condition; RME, *n* = 12 plates per condition, each with 7 stimulations per recording (only the first stimulation is shown). Two-sided Wilcoxon rank-sum test with Bonferroni correction (AUA, *P* = 0.0019; RME, *P* = 0.0001). Data are mean ± 95% CI. **e**, Number of reorientation starts per minute in animals expressing the optogenetic silencing channel *GtACR2* under cell-specific promoters for AUA and RME. Two-sided Wilcoxon rank-sum test with Bonferroni correction (AUA, *P* = 0.16; RME, *P* = 0.178). AUA, *n* = 15 −ATR, 16 +ATR plates; RME, *n* = 15 −ATR, 14 +ATR plates. Data show median, IQR and range. **f**, SIAV activity as animals encounter the aversive odor octanol (orange). Neuron activity during similar length epochs of spontaneous forward movement (gray), shown as a control for how these cells’ activities change with locomotion (for example, consider **a**). The vertical gray dashed line shows octanol encounter. *n* = 85 odor epochs, 163 spontaneous epochs. Two-sided Wilcoxon rank-sum test comparing average activity on octanol and during spontaneous movement (*P* = 0.0134), data are mean ± 95% CI. **g**, AUA, RME and RIB activity as animals encounter octanol in orange, control spontaneous movement in gray, as in **f**. None of the comparisons are significant using a two-sided Wilcoxon rank-sum test with Bonferroni correction comparing average activity on octanol and during spontaneous movement. Data are mean ± 95% CI. **h**, SIAV activity as animals exit diacetyl in red. Spontaneous movement is shown as a control for how these cells’ activities change with locomotion. The vertical gray dashed line shows the moment of diacetyl exit. Two-sided Wilcoxon rank-sum test comparing average activity off diacetyl and during spontaneous movement (*P* = 0.0173). Data are mean ± 95% CI. **i**, AUA, RME and RIB activity as animals exit diacetyl is shown in red, with similar off odor movement shown in gray, as in **h**. Two-sided Wilcoxon rank-sum test with Bonferroni correction comparing average activity off diacetyl and during spontaneous movement (AUA, *P* = 0.0142). Data are mean ± 95% CI. **j**, Fraction animals making the correct dorsal versus ventral turn in a butanone gradient in animals expressing *GtACR2* under the previously described cell-specific promoters for AUA, RME and SIA. Only reversals that end during the optogenetic inhibition were included in this analysis. Each dot is one plate with 20–100 animals, black dots show data mean. *n* = 11–14 recordings. None of the comparisons are significant with a two-sided Wilcoxon rank-sum test with Bonferroni correction. **k**, Reorientation rate compared to bearing to odor during butanone chemotaxis for SIA::GtACR2 animals. *n* = 15 recording plates per condition. Two-sided Wilcoxon rank-sum test comparing slopes of the rate of reorientation initiation (*P* < 0.0001). Data show mean ± 95% CI. For all panels, significance is noted as: NS (not significant), *P < 0.05, **P < 0.01, ***P < 0.001 and **P < 0.0001. For panels with multiple comparisons, symbols denote Bonferroni-adjusted *P* values.

**Extended Data Fig. 4:**
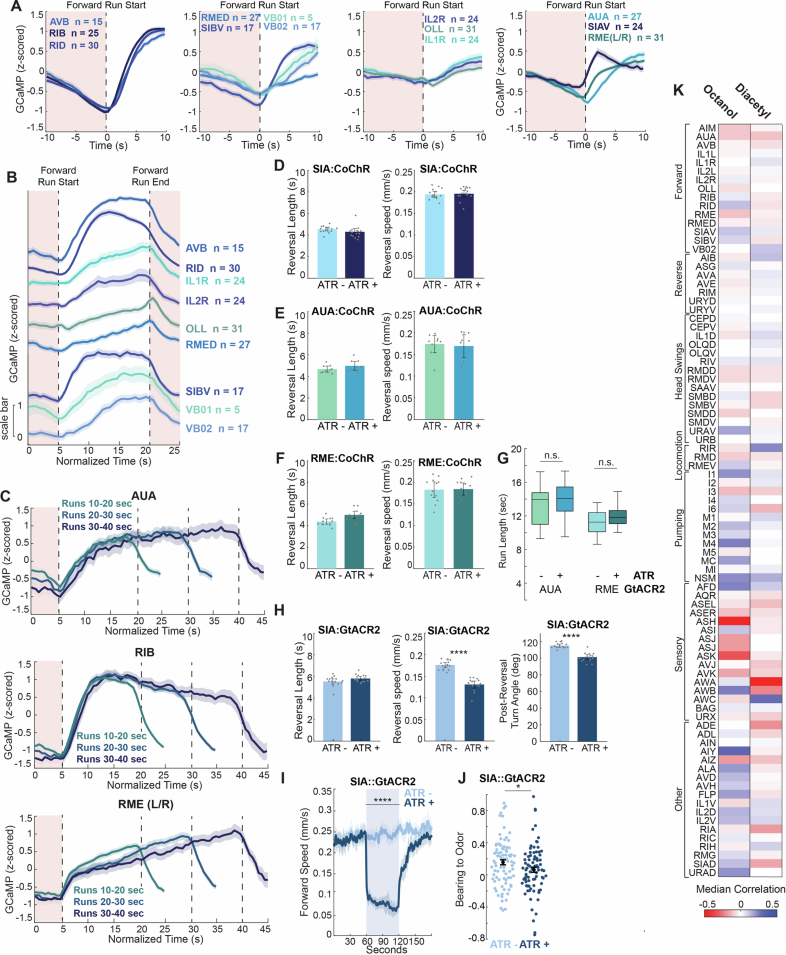
Further analysis of the forward-reverse neurons. **a**, Z-scored neuron activity aligned to times when animals switch from reverse to forward movement. Dashed black line shows forward run start, red shading is during the reversal. n = 175–762 runs (n values on the plot show the number of recordings with data for that neuron). Data are mean ± 95% CI. **b**, Neuron activity across entire forward runs, as in Fig. 4a. Data are from runs that were 10–20 s, and z-scored neuron activity during the run was uniformly compressed or expanded to align with a standard 15 s run. Dashed black lines show run start and end; red shading is during reversals. n values on the plot show the number of recordings with data for that neuron. Data are mean ± 95% CI. **c**, AUA, RIB, and RME(L/R) activity across different run lengths. Data are from runs of the indicated lengths; activity is uniformly compressed or expanded to align with a uniform 15, 25, or 35 s run as indicated, similar to **b**. n = 34–272 runs. Data are mean ± 95% CI. **d**–**f**, Effects on reversal length and speed of optogenetic activation of SIA, AUA, or RME using the blue light activated CoChR opsin. SIA n = 14 −ATR and 15 +ATR recordings. AUA n = 9 plates per condition. RME n = 12 plates per condition. For all genotypes, there are 7 stimulations per recording plate. Two-sided Wilcoxon rank-sum test with Bonferroni correction (none of these comparisons are significant). Data are mean ± 95% CI. **g**, Forward run length in seconds in animals expressing *GtACR2* under each of the previously described cell-specific promoters for AUA and RME. Only runs that start during the optogenetic inhibition were included in this analysis. None of the comparisons is significant with a two-sided Wilcoxon rank-sum test with Bonferroni correction. n = AUA::Gt2 no ATR 11 recordings, AUA::Gt2 ATR 12 recordings, RME Gt2 13–14 recordings. Data show median, IQR, and range. **h**, From left to right, effects on reversal length, speed, and postreversal turn angle during optogenetic inhibition of SIA. n = 15 recording plates per condition. Two-sided Wilcoxon rank-sum test with Bonferroni correction, comparing average values during stimulation for each recording (*p* < 0.0001, *p* < 0.0001). Data are mean ± 95% CI. **i**, Forward speed during optogenetic inhibition of SIA. n = 15 recording plates per condition, 6 optogenetic stimulations per recording. Two-sided Wilcoxon rank-sum test, comparing average speed with and without ATR (*p* < 0.0001). Data are mean ± 95% CI. **j**, Bearing at pirouette end during butanone chemotaxis in SIA::GtACR2 animals. n = 15 recorded plates in each condition, 6 stimulations per recording, only pirouettes that end during the optogenetic stimulus were included in this analysis. Two-sided Wilcoxon rank-sum test (*p* = 0.0334). Data show mean ± s.e.m. **k**, Correlations of all recorded neurons to the sensory neuron for each odor, AWA for diacetyl (right) and ASH for octanol (left). Colored based on the median Pearson correlation coefficient for all videos with both neurons recorded; red shows a negative correlation, blue shows positive. Neurons are divided into functional groups based on activity, as determined in this study and in ref. ^15^. ‘Locomotion’ neurons clearly associate with an animal’s movement state, but not exclusively forward or reverse movement. ‘Other’ neurons did not show clear associations with any of the listed categories in our data. For all panels, significance is noted as: NS (not significant), *P < 0.05, **P < 0.01, ***P < 0.001 and **P < 0.0001. For panels with multiple comparisons, symbols denote Bonferroni-adjusted *P* values.

Our next goal was to identify sensory-responsive neurons that regulate transitions between forward and reverse movement, another key feature of olfactory navigation (Fig. 1c,d,k,l). Using our brain-wide calcium imaging data, we first focused on identifying all neurons with higher activity during spontaneous forward movement (Extended Data Fig. 4a,b). Consistent with past work^15,41,43,44^, the AVB, RIB and RID neurons activate as forward movement begins and maintain high activity during runs (Extended Data Fig. 4a). We also identified a large set of other forward-active neurons with different dynamics during forward movement (Fig. 4a and Extended Data Fig. 4a,b). We thus focused on a few cells with distinct activity patterns—AUA, RME and SIAV. For example, RME activity increases monotonically throughout forward runs (Fig. 4a and Extended Data Fig. 4c), while SIAV activates at run starts and decays thereafter (Fig. 4a).

These divergent dynamics among forward-active cells suggested possible differences in their function. We therefore tested how these neurons impact spontaneous behavior. We expected optogenetic activation of these forward-active cells to promote fast-forward movement, as has been seen for the forward-active cells AVB, RIB and RID^43–45^. Indeed, optogenetically activating the SIAs increased forward speed (Fig. 4b and Extended Data Fig. 4d). By contrast, activating RME or AUA significantly increased reversal frequency (Fig. 4c,d) but not length or speed (Extended Data Fig. 4e,f), suggesting RME and AUA trigger reversal initiation. Notably, silencing AUA or RME did not affect reversal probability (Fig. 4e) or forward run length (Extended Data Fig. 4g), suggesting these cells are sufficient but not necessary for reversal starts. Silencing the SIAs resulted in slower forward movement, as previously seen^46^, and also caused slower reversals and lower-angle turns (Extended Data Fig. 4h,i), suggesting the SIAs bias the animal toward faster movement and forward movement.

We then asked whether these neurons show odor-modulated activity. Notably, SIAV activity decreased as animals approached the aversive odor octanol and remained suppressed throughout the octanol encounter (Fig. 4f). In contrast, the other forward-active neurons showed no sensory-responsive signals (Fig. 4g). SIAV activity also significantly decreased when leaving diacetyl (Fig. 4h,i). This suggests that SIAV activity declines when animals encounter sensory conditions that bias them away from forward movement.

We then examined whether any of these cells had a role in navigation. We found that directed turns were intact when either RME, AUA or SIAs were silenced (Fig. 4j). However, animals with the SIAs silenced began reorientations (Fig. 4k) and forward runs (Extended Data Fig. 4j) in a worse direction in the odor gradient, suggesting that SIA’s control over forward-reverse transitions contributes to odor-guided reversals and runs.

Finally, to cast a wide net, we examined correlations between all recorded neurons and the sensory neurons of interest—ASH for octanol recordings and AWA for diacetyl. Other sensory neurons (AWB, AWC, etc.) showed opposite correlations with AWA and ASH, suggesting that they may encode the valence of sensory cues (Extended Data Fig. 4k). Feeding neurons were anticorrelated with ASH, which may prompt animals to eat less in an aversive environment. Notably, several neurons that did not show strong associations with behavior had strong correlations with ASH or AWA, suggesting a role in sensory processing (Extended Data Fig. 4k).

### Tyramine is required for sensory-guided reorientations during navigation

**Fig. 5:**
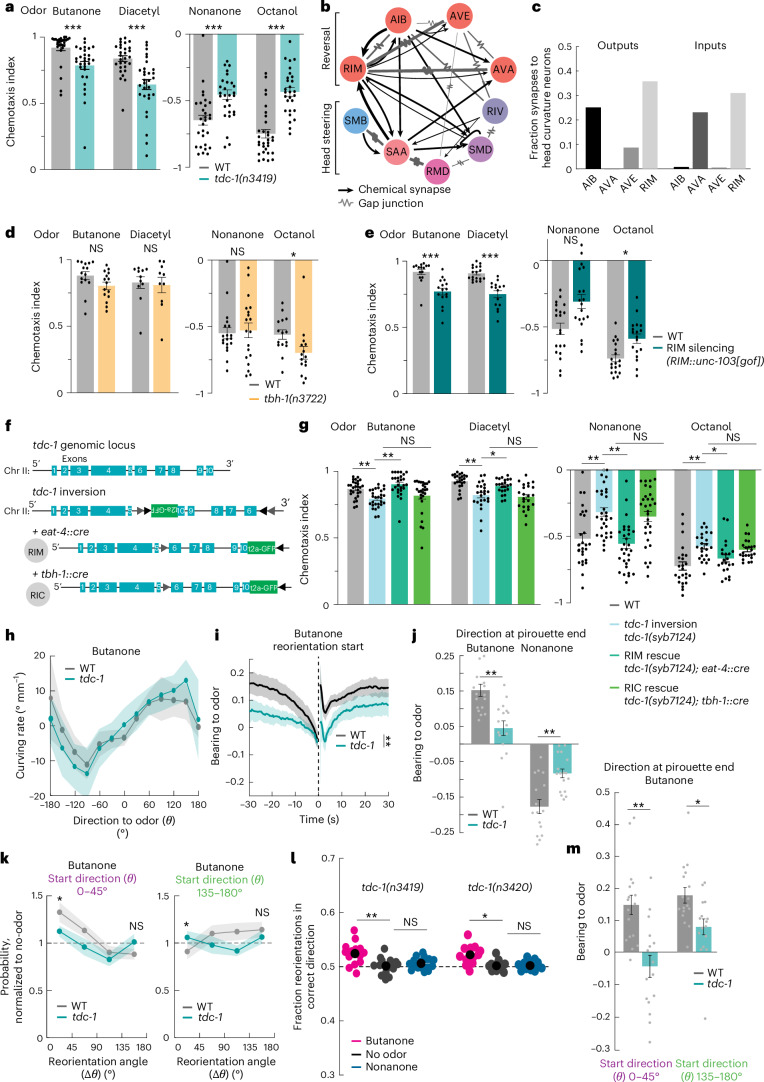
RIM tyramine is required for sensory-guided reorientations during olfactory navigation. **a**, Chemotaxis of WT and *tdc-1(n3419)* animals to the attractive odors butanone and diacetyl and the aversive odors nonanone and octanol. Chemotaxis index is calculated as (number of animals at odor − number of animals at ethanol control)/(total number of animals). From left to right, *n* = 28, 30, 29, 30, 28, 28, 31 and 28 plates. Mann–Whitney *U* test with Bonferroni correction (from left to right, *P* = <0.0001, <0.0001, 0.0001, <0.0001). Data show mean ± s.e.m., each dot shows one plate. **b**, Connectivity between the canonical reverse-promoting neurons (top neurons) and the head-steering circuit (bottom neurons). Data from ref. ^12^. **c**, Fraction of synapses between each of the reverse-promoting neurons and the head-steering circuit neurons in **b** showing both outputs and inputs. Electrical synapses were counted as both inputs and outputs. Data from ref. ^12^. **d**, Chemotaxis of WT and *tbh-1(n3722)* animals. From left to right, *n* = 15, 15, 11, 10, 19, 19, 15 and 15 plates. Mann–Whitney *U* test with Bonferroni correction (from left to right, *P* = 0.041, 0.9725, 0.773, 0.006). Data show mean ± s.e.m. **e**, Chemotaxis of WT and RIM silenced animals. RIM silencing is the intersection of *tdc-1::cre* *+* *glr-1::inv(unc-103[gof])*. From left to right, *n* = 18, 15, 15, 16, 20, 19, 19 and 18 plates. Mann–Whitney *U* test with Bonferroni correction (from left to right, *P* = <0.0001, <0.0001, 0.015, 0.003). Data show mean ± s.e.m. **f**, CRISPR–Cas9 editing of the endogenous genome at *tdc-1* was used to make a conditional rescue allele of *tdc-1* for rescue in cell(s) of interest (Methods). **g**, Chemotaxis of WT animals, *tdc-1* mutants with CRISPR inversion, and conditional rescues in RIM through *eat-4::cre* expression or RIC through *tbh-1::cre* expression. From left to right, *n* = 28, 28, 27, 27, 24, 24, 23, 23, 28, 31, 31, 30, 24, 24, 22 and 23 plates. Mann–Whitney *U* test with Bonferroni correction (from left to right, *P* = <0.0001, <0.0001, 0.025, <0.0001, 0.0048, 0.5163, 0.0002, <0.0001, 0.76, 0.001, 0.0032, 0.39). **h**, Weathervaning behavior of WT and *tdc-1(n3419)* animals, as in Extended Data Fig. 1b. Data show mean ± 95% CI. **i**, Average bearing to odor over time of WT and *tdc-1(n3419)*, as in Fig. 1d. Two-sided Wilcoxon rank-sum test comparing the pre-reversal slopes of bearing over time (*P* = 0.003). *n* = 16–17 recordings. Data are mean ± 95% CI. **j**, Bearing to odor at the ends of pirouettes, as in Fig. 1k. From left to right, *n* = 17, 16, 16 and 17 recordings. Two-sided Wilcoxon rank-sum test with Bonferroni correction (*P* = 0.0005 and 0.0021). Data are mean ± s.e.m. **k**, Change in direction (∆*θ*) executed by animals that start with a small (left, purple) or large (right, green) angle direction to the odor (*θ*), normalized to no-odor controls of the same genotype. Two-sided Wilcoxon rank-sum test with Bonferroni correction (from left to right, *P* = 0.0065, *P* = 0.0136, *P* = 0.0033, *P* = 0.126). *n* = 16–17 recordings. Data show mean ± 95% CI. **l**, Fraction of reorientations that turn the animal in the correct dorsal or ventral direction, comparing *tdc-1(n3419)* (left) and *tdc-1(n3420)* (right) to the same genotype no-odor controls. Two-sided Wilcoxon rank-sum test with Bonferroni correction. *n* = 16–18 recording plates. Black dots show data mean. **m**, Bearing at the ends of pirouettes, separated by if the animal was facing away from the odor or toward the odor when the reorientation at the end of the pirouette began. Two-sided Wilcoxon rank-sum test with Bonferroni correction. *n* = 17 WT and 16 *tdc-1* recordings. Data are mean ± s.e.m. For all panels, significance is noted as: NS (not significant), *P < 0.05, **P < 0.01, ***P < 0.001 and **P < 0.0001. For panels with multiple comparisons, symbols denote Bonferroni-adjusted *P* values.

**Extended Data Fig. 5:**
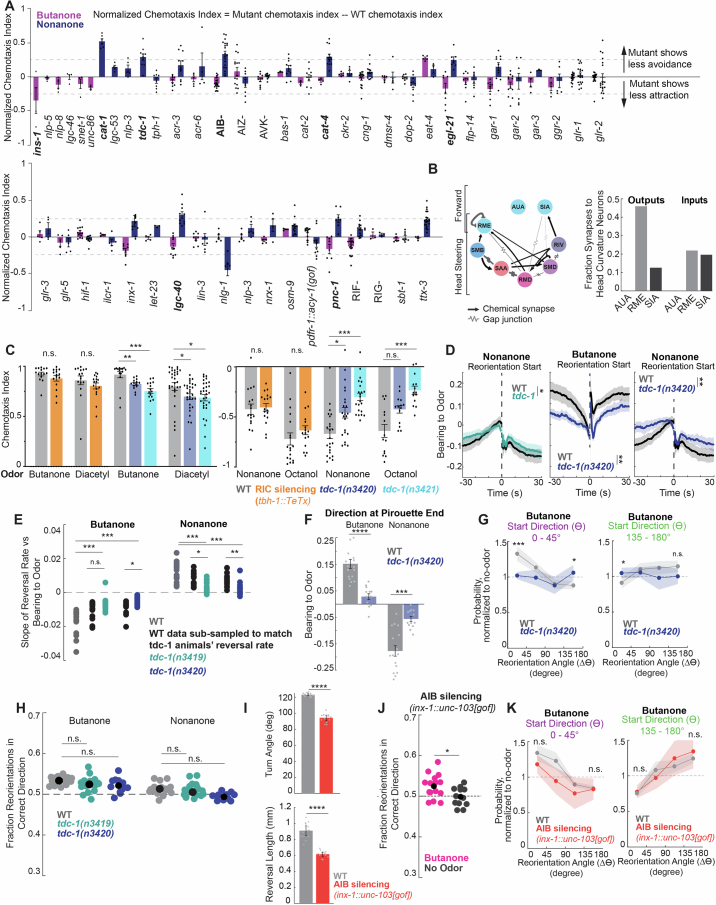
Candidate genetic screen for navigation mutants and additional analyses related to tyramine signaling. **a**, Chemotaxis screen, examining responses to the attractive odor butanone (shown in pink) and the aversive odor nonanone (shown in blue). Chemotaxis indices are normalized to wild-type controls run on the same day (that is, the average wild-type chemotaxis index for that odor from that day is subtracted from the chemotaxis index from each mutant plate run the same day). Therefore, if mutant strains have a chemotaxis deficit compared to wild-type animals, their normalized chemotaxis index will be positive for nonanone, and it will be negative for butanone. In the x-axis labels, neuron silencing lines are in capital letters, and endogenous mutations are in italics. Alleles and strains used are listed in the strain table. Strains where the difference between mutant and wild type chemotaxis is >0.25 are in bold. n = 3–21 plates over 1+ days with 50–200 animals per plate. n values: *ins-1* = 4; *nlp-5* = 4; *nlp-8* = 4; *lgc-46* = 4; *snet-1* = 4; *unc-86* = 3; *cat-1* = 6; *lgc-53* = 4; *nlp-3* = 5; *tdc-1* = 10; *tph-1* = 8; *acr-3* = 10 per odor; *acr-6* = 5 butanone, 4 nonanone; AIB silencing = 20 each odor; AIZ silencing = 20 and 15; AVK silencing = 10 per odor; *bas-1* = 3 and 10; *cat-2* = 3 and 10; *cat-4* = 4 and 13; *ckr-2* = 5 per odor; *cng-1* = 19 and 13; *dmsr-4* = 5 per odor; *dop-2* = 9 and 10; *eat-4* = 5 and 3; *egl-21* = 8 and 7; *flp-14* = 9 and 8; *gar-1* = 10 each odor; *gar-2* = 12 and 7; *gar-3* = 4 and 3; *ggr-2* = 5 per odor, *glr-1* = 19 per odor; *glr-2* = 19 per odor; *glr-3* = 5 and 4; *glr-5* = 5 per odor; *hif-1* = 13 and 10; *ilcr-1* = 5 each odor; *inx-1* = 9 and 10; *let-23* = 4 and 3; *lgc-40* = 10 per odor; *lin-3* = 9 and 6; *nlg-1* = 6 per odor; *nlp-3* = 5 per odor; *nrx-1* = 5 and 4; *osm-9* = 5 and 13; *pdfr-1::acy-1[gof]* = 14 and 11; *pnc-1* = 8 and 7; RIF silencing = 17 and 15; RIG silencing = 3 per odor; *sbt-1* = 7 and 8; *ttx-3* = 6 and 20. Note that not every mutant was tested to both odors. **b**, Left, connectivity of the forward-associated neurons studied here (top neurons) and the head-steering circuit (bottom; data from ref. ^12^). As RME sends synaptic outputs to the head and neck muscles, it can be considered both a forward and a head-steering neuron, as indicated. Right, fraction of synapses between each of the forward-associated neurons and any of the head-steering circuit neurons (defined as those shown in the circuit diagram), showing both outputs and inputs to each forward-promoting neuron. Electrical synapses were counted as both inputs and outputs. **c**, Chemotaxis of wild type and RIC silenced animals (*tbh-1::TeTx*) as well as separate wild type controls and two other alleles of *tdc-1* (*n3420* and *n3421*) to the attractive odors butanone and diacetyl and the aversive odors nonanone and octanol. Chemotaxis index is calculated as (number of animals at odor − number of animals at ethanol (control))/(total number of animals). Attractive odor n values, from left to right: 15, 15, 16, 16, 15, 14, 15, 30, 30, 31. Aversive odor n values, from left to right: 17, 17, 18, 16, 23, 23, 22, 14, 14, 14. Experiments were run over 3+ days with 50–200 animals per plate. Mann–Whitney *U* test with Bonferroni correction (p values: attractive, from left to right: 0.233, 0.051, 0.0005, 0.0001, 0.0015, 0.0016. Aversive, from left to right: 0.557, 0.088, 0.0042, <0.0001, 0.0205, <0.0001). **d**, Average bearing to odor aligned to reorientation start times during butanone or nonanone chemotaxis. The dashed line shows reversal start and end. Two-sided Wilcoxon rank-sum test with Bonferroni correction comparing the pre-reversal slopes of bearing over time (from left to right, *p* = 0.005, *p* = 0.0004, *p* = 0.0005). n = 16–18 recording plates. Data are mean ± 95% CI. **e**, Relationship between bearing to odor and reorientation rates in WT and *tdc-1* animals. This was quantified as the slope of the reversal start vs bearing to odor plot for each recording. In this case, because *tdc-1* animals are less likely to reverse, we wanted to perform a control analysis to examine how a reduced reversal rate would impact these results. Therefore, we randomly removed reversals from wild-type data so that they reversed at the same rate as the comparison *tdc-1* genotype (shown in black). Each dot is a single recording. n = 16–18 recording plates. Two-sided Wilcoxon rank-sum test with Bonferroni correction (from left to right, butanone: *p* < 0.0001, *p* = 0.155, *p* < 0.0001, *p* = 0.003; nonanone, *p* = 0.0002, *p* = 0.007, *p* < 0.0001, *p* = 0.0013). **f**, Bearing to odor at the ends of pirouettes during butanone or nonanone chemotaxis. From left to right, n = 17, 12, 16, 13. Two-sided Wilcoxon rank-sum test with Bonferroni correction (from left to right, *p* < 0.0001, *p* = 0.0002). Data are mean ± s.e.m. **g**, Change in direction (∆*θ*) executed by wild type or *tdc-1(n3420)* animals that start with a small (left, purple) or large (right, green) angle direction to the odor (*θ*), normalized to no odor controls. *tdc-1* animals do not modulate turn amplitudes as much as WT animals do. Two-sided Wilcoxon rank-sum test with Bonferroni correction (from left to right, *p* = 0.0001, *p* = 0.0043, *p* = 0.0056, *p* = 0.025). n = 16–18 recording plates. Data show mean ± 95% CI. **h**, Fraction of reorientations that turn the animal in the correct dorsal or ventral direction, comparing wild type, *tdc-1(n3419)*, and *tdc-1(n3420)*. Note that although wild-type animals and *tdc-1* animals are not significantly different, *tdc-1* animals do not show a difference in the fraction of correct turns when comparing their own spontaneous and nonanone reorientations (Fig. 5). Each dot is one plate with 20–100 animals. None of the comparisons is significant using a two-sided Wilcoxon rank-sum test with Bonferroni correction. n = 16–18 recordings. Black dots show data mean. **i**, Reversals are shorter and smaller angle in AIB-silenced animals. AIB silencing is *inx-1::unc-103[gof]*. Top, graph compares the absolute value of postreversal turn angle in wild-type and AIB-silenced animals; bottom, compares reversal length. Two-sided Wilcoxon rank-sum test with Bonferroni correction (both *p* < 0.0001). n = 12 AIB silenced and 13 wild-type recording plates. Data show mean ± 95% CI. **j**, Fraction of reorientations that turn the animal in the correct dorsal or ventral direction, comparing AIB silencing *(inx-1::unc-103[gof])* animals in a butanone gradient to no odor movement of the same genotype. Two-sided Wilcoxon rank-sum test (*p* = 0.03). n = 12–15 recording plates. Black dots show data mean. **k**, Change in direction (∆*θ*) executed by wild type or AIB silencing *(inx-1::unc-103[gof])* animals that start with a small (left, purple) or large (right, green) angle direction to the odor (*θ*), normalized to no odor controls. None of the comparisons is significant using a two-sided Wilcoxon rank-sum test with Bonferroni correction. n = 12–15 recording plates. Data show mean ± 95% CI. For all panels, significance is noted as: NS (not significant), *P < 0.05, **P < 0.01, ***P < 0.001 and **P < 0.0001. For panels with multiple comparisons, symbols denote Bonferroni-adjusted *P* values.

The above results suggest that neural dynamics evolve in a stereotyped manner during each reorientation. We next sought to identify the neurons that control these reorientation-associated brain dynamics. Briefly, we performed a chemotaxis assay screen of >50 mutants defective in cell specification, neurotransmission and neuromodulation (Extended Data Fig. 5a). From this screen, we identified *tdc-1* mutants as deficient in chemotaxis to attractive and aversive odors (Fig. 5a). Thus, *tdc-1* is required for the production of the neurotransmitter tyramine, produced by RIM neurons (and non-neuronal sources), and octopamine, which is synthesized in RIC neurons using tyramine as a precursor^47^. RIM is densely connected to the head-steering circuit (Fig. 5b,c and Extended Data Fig. 5b), although tyramine receptors are also found in neurons without synaptic input from RIM^48^.

We performed additional genetic and cell silencing experiments to test whether RIM tyramine is specifically required for chemotaxis. First, we confirmed that three independent mutant alleles of *tdc-1* all shared the chemotaxis deficit (Extended Data Fig. 5c). By contrast, animals lacking *tbh-1*, the enzyme that converts tyramine into octopamine^47^, or with the octopaminergic neuron RIC silenced, had navigation comparable to WT (Fig. 5d and Extended Data Fig. 5c). This suggests that RIC activity and octopamine are dispensable for navigation. Silencing the neuron RIM led to deficient chemotaxis to most odors tested (Fig. 5e). Finally, we used CRISPR–Cas9 genome editing to create a conditional rescue allele of *tdc-1* (Fig. 5f). As expected, the inverted strain had defective chemotaxis. Restoring expression in RIM, but not RIC, resulted in WT chemotaxis to all odors tested (Fig. 5g). Together, these experiments suggest that tyramine release from RIM is critical for olfactory navigation.

We then sought to identify the exact behaviors that tyramine influences during chemotaxis. We compared the navigation strategies of WT animals to two different *tdc-1* mutant alleles and observed several deficits shared by both mutants. Weathervaning was unaffected in the absence of tyramine (Fig. 5h), but *tdc-1* animals were less likely to bias their reversal starts based on their heading in the odor gradient (Fig. 5i and Extended Data Fig. 5d,e). Additionally, *tdc-1* animals began forward runs in less favorable gradient directions (Fig. 5j and Extended Data Fig. 5f). This was likely related to their inability to modulate the amplitudes of their reorientation-associated turns based on the olfactory gradient (Fig. 5k and Extended Data Fig. 5g). *tdc-1* mutants also had a partial deficit in modulating the D/V direction of their turns (Fig. 5l and Extended Data Fig. 5h).

We were concerned that some of these sensory turning deficits could be due to the well-documented changes in reversal properties of *tdc-1* mutants. These animals have shorter reversals and reduced turn amplitudes^47,49–52^. Specifically, we were concerned that animals lacking tyramine might not navigate well due to executing lower-angle turns. However, *tdc-1* animals began forward runs in worse directions than WT animals whether they began facing toward or away from the odor (Fig. 5m). Additionally, compared to WT, animals lacking tyramine were less likely to make low-angle turns when they had low-angle errors in their odor bearing and more likely to make low-angle turns when they had high-angle errors (Fig. 5k). Finally, animals with the reversal neuron AIB silenced, which have deficits in reversal length and angle^33^ (Extended Data Fig. 5i), had intact D/V correctness and amplitude modulation of their odor-guided reorientations (Extended Data Fig. 5j,k). This suggests that disrupting reorientations does not always affect directed turning. Together, these data show that tyramine is necessary to properly time and execute error-correcting reorientations during chemotaxis.

### RIM tyramine controls reorientations through parallel pathways

**Fig. 6:**
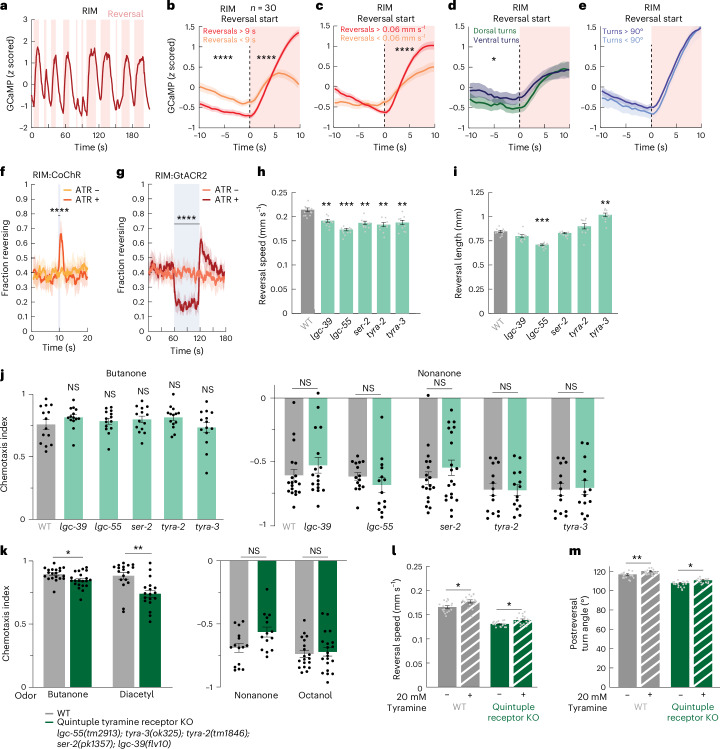
RIM tyramine influences reorientations through multiple parallel pathways. **a**, RIM GCaMP trace from a single animal. **b**, RIM activity aligned to reversal starts (dashed line, reversal is shaded red), split by reversal length. Two-sided Wilcoxon rank-sum test with Bonferroni correction, comparing activity before (*P* < 0.0001) and after (*P* < 0.0001) reversal start. *n* = 738 reversals (*n* values on the plot show the number of recordings with data for RIM). Data show mean ± 95% CI. **c**, RIM activity as in **b**, with reversals split by reversal speed. Two-sided Wilcoxon rank-sum test with Bonferroni correction, comparing before (*P* = 0.65) and after (*P* < 0.0001) reversal start. *n* = 738 reversals. Data show mean ± 95% CI. **d**, RIM activity as in **b**, split by direction of the postreversal turn, either dorsal or ventral. Reversals are subsampled to match length and speed. Two-sided Wilcoxon rank-sum test with Bonferroni correction, separately comparing activity before (*P* = 0.0015) and after (*P* = 0.15) reversal start. *n* = 148 dorsal turn and 163 ventral turn reversals. Data show mean ± 95% CI. **e**, RIM activity as in **b**, split by postreversal turn angle. Reversals are subsampled to match length and speed. Two-sided Wilcoxon rank-sum test with Bonferroni correction. *n* = 74 reversals. Data show mean ± 95% CI. **f**, Fraction of animals reversing, with the shading showing optogenetic activation of RIM. Animals are *tdc-1::cre* *+* *glr-1::inv(CoChR)*. *n* = 12–15 recording plates, 10 optogenetic stimulations per recording (only the first stimulation is shown here). Two-sided Wilcoxon rank-sum test (*P* < 0.0001). Data are mean ± 95% CI. **g**, Fraction of animals reversing across time, with the blue bar showing optogenetic inhibition of RIM. Animals are *tdc-1::cre* *+* *glr-1::inv(GtACR2)*. *n* = 11–14 recording plates, 3 optogenetic stimulations per recording (only the first stimulation is shown here). Two-sided Wilcoxon rank-sum test (*P* < 0.0001). Data are mean ± 95% CI. **h**, Reversal speed for WT animals and animals lacking each of the five known tyramine receptors. Animals were off food without odor. *n* = 10 recording plates per genotype. Two-sided Wilcoxon rank-sum test with Bonferroni correction, comparing each mutant to WT (from left to right, *P* = 0.001, 0.00018, 0.0006, 0.0003, 0.0017). Data are mean ± 95% CI. **i**, Reversal length for WT animals and animals lacking each of the five known tyramine receptors. *n* = 10 recording plates per genotype. Two-sided Wilcoxon rank-sum test with Bonferroni correction, comparing each mutant to WT (*P* = 0.00018 and 0.0002). Data are mean ± 95% CI. **j**, Chemotaxis of WT animals and animals lacking each of the five known tyramine receptors. None of the comparisons is significant with a Mann–Whitney *U* test with Bonferroni correction. For butanone, *n* = 14 plates per genotype. For nonanone, from left to right, *n* = 19, 17, 15, 14, 19, 20, 14, 14, 14, 14 plates. Data show mean ± s.e.m. **k**, Chemotaxis of WT animals and quintuple mutant animals lacking all of the five known tyramine receptors. From left to right, *n* = 20, 21, 18, 18, 15, 14, 19, 20 plates. Mann–Whitney *U* test with Bonferroni correction (from left to right, *P* = 0.006, 0.0006, 0.05, 0.83). Data show mean ± s.e.m. **l**, Reversal speed for WT animals and quintuple mutant animals lacking all of the five known tyramine receptors. Animals were off food without odor. From left to right, *n* = 16, 16, 16 and 18 plates. Two-sided Wilcoxon rank-sum test with Bonferroni correction (*P* = 0.011 and 0.01). Data are mean ± 95% CI. **m**, Postreversal turn angle as in **l**. Two-sided Wilcoxon rank-sum test with Bonferroni correction (*P* = 0.005 and 0.0127). Data are mean ± 95% CI. For all panels, significance is noted as: NS (not significant), *P < 0.05, **P < 0.01, ***P < 0.001 and **P < 0.0001. For panels with multiple comparisons, symbols denote Bonferroni-adjusted *P* values.

**Extended Data Fig. 6:**
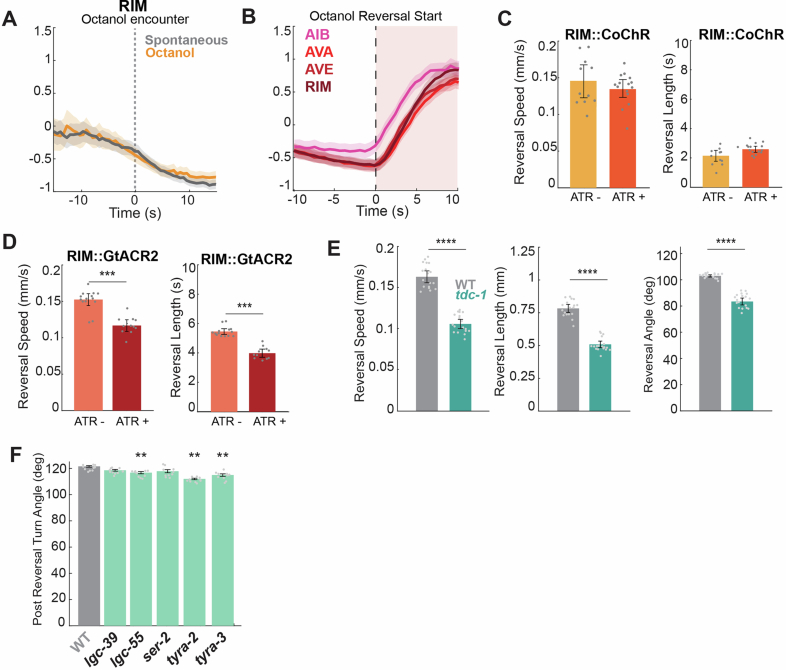
Additional behavioral effects of RIM and tyramine signaling perturbations. **a**, RIM activity is unaffected when animals move forward onto octanol. Dashed gray line shows octanol encounter. Spontaneous forward movement epochs are sampled to be a similar length of forward movement as octanol encounters, to allow a comparison of RIM activity during spontaneous forward movement versus forward movement onto octanol. Two-sided Wilcoxon rank-sum test compares spontaneous and octanol activity (comparison is not significant). Data are mean ± 95% CI. **b**, Reversal neuron activity during octanol-triggered reversals (defined as any reversals that begin with the animal’s head on octanol). Dashed line shows reversal start, red shading shows the reversal. Data are mean ± 95% CI. **c**, Reversal length and speed during optogenetic RIM activation or during spontaneous reversals on no-ATR plates. n = 12 −ATR and 15 +ATR recording plates. Two-sided Wilcoxon rank-sum test with Bonferroni correction (comparison is not significant). Data are mean ± 95% CI. **d**, Reversal speed and length during optogenetic RIM inhibition or during spontaneous reversals on no-ATR plates. n = 14 −ATR and 11 +ATR recording plates. Two-sided Wilcoxon rank-sum test with Bonferroni correction (left *p* = 0.0002, right *p* < 0.0001). Data are mean ± 95% CI. **e**, Reversal speed, length, and postreversal turn angle for wild type and *tdc-1(n3419)* animals. Animals are off food without odor. n = 18 recordings per genotype. Two-sided Wilcoxon rank-sum test with Bonferroni correction (all three *p* < 0.0001). Data are mean ± 95% CI. **f**, Postreversal turn angle for wild-type animals and animals lacking each of the five known tyramine receptors. Animals were off food without odor. n = 10 recording plates per genotype. Two-sided Wilcoxon rank-sum test with Bonferroni correction comparing mutant turn angles to wild type (from left to right, *p* = 0.014, *p* = 0.0006, *p* = 0.054, *p* = 0.0002, *p* = 0.0004). Data are mean ± 95% CI. For all panels, significance is noted as: NS (not significant), *P < 0.05, **P < 0.01, ***P < 0.001 and **P < 0.0001. For panels with multiple comparisons, symbols denote Bonferroni-adjusted *P* values.

We next investigated how RIM activity and tyramine release impact sensory-guided behavior. RIM is a well-established reversal-active neuron^41^, with high activity throughout reversals regardless of postreversal turn direction (Fig. 6a–e). RIM activity was unaffected as animals encountered octanol (Extended Data Fig. 6a), although it activated once animals initiated reversals on octanol (Extended Data Fig. 6b). Consistent with past work^52,53^, we found that optogenetically activating RIM promoted reversal initiation (Fig. 6f and Extended Data Fig. 6c). Inhibiting RIM led to fewer, shorter, slower reversals (Fig. 6g and Extended Data Fig. 6d), similar to *tdc-1* animals (Extended Data Fig. 6e). Together, these results are consistent with a reversal-promoting effect for RIM.

RIM releases tyramine as well as glutamate and neuropeptides^52,54^. To focus on the downstream targets of tyramine in particular, we examined the tyramine receptors. Five tyramine receptors are known—LGC-39, LGC-55, SER-2, TYRA-2 and TYRA-3 (refs. ^55,56^). To examine the contribution of each receptor to reorientation behaviors, we compared the spontaneous behavior of WT to mutants lacking single tyramine receptors. SER-2 promotes high-angle turns^49^ and LGC-55 promotes reversal length^51^. We further observed that all of the receptors impact reversal speed, four impact turn angles and several others influence reversal termination (Fig. 6h,i and Extended Data Fig. 6f). These results suggest tyramine acts through each receptor nonredundantly to control reorientation behaviors.

We then considered which of these receptors had a role in navigation. Animals with single tyramine receptor mutations showed no chemotaxis deficits (Fig. 6j). Quintuple mutants with mutations in all five receptors showed deficient responses to some, but not all, odors (Fig. 6k). Thus, the quintuple mutant phenotype is less severe than all three *tdc-1* mutant strains. One possible explanation for this discrepancy is that there is a remaining, unidentified tyramine receptor. To investigate this possibility, we quantified the behavior of WT animals and quintuple mutants lacking all known tyramine receptors, comparing behavior with or without exogenous tyramine. As previously shown^51^, the addition of tyramine resulted in faster, higher-angle reorientations in WT animals (Fig. 6l,m). Exogenous tyramine also affected reorientations in the quintuple mutant (Fig. 6l,m), suggesting the presence of one or more other unidentified receptor(s). However, we cannot rule out whether this was due to the conversion of tyramine to excess octopamine. Overall, these results suggest that tyramine likely acts in parallel on several receptors to modulate locomotion and navigation.

### Neurons that direct behavioral sequences are broadly dysregulated in *tdc-1* animals

**Fig. 7:**
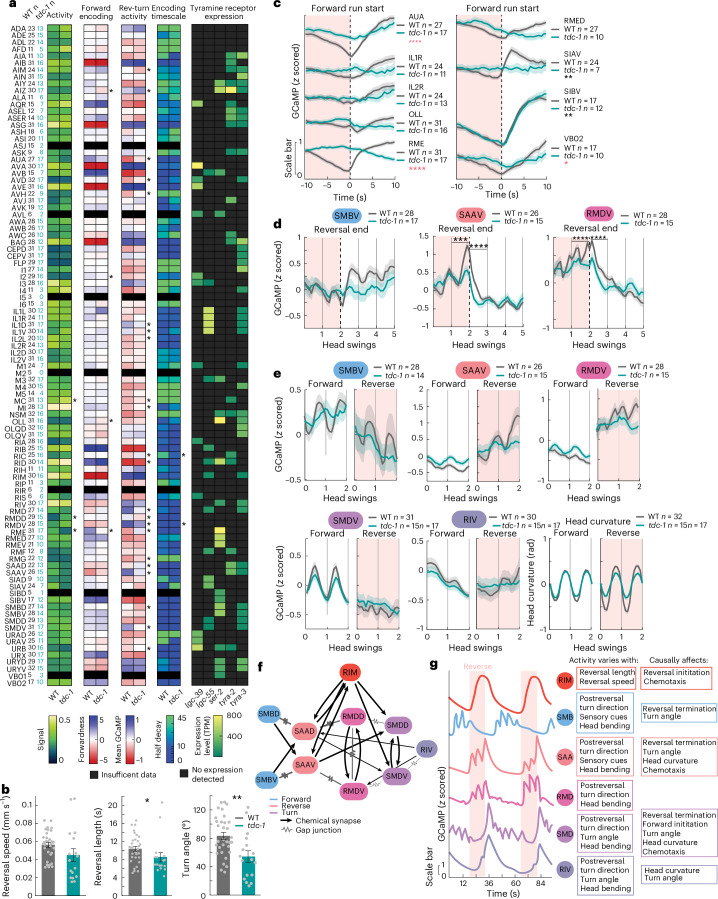
Neurons that direct reorientation behaviors are broadly dysregulated in *tdc-1* mutant animals. **a**, Comparisons of activity features across neurons in WT and *tdc-1* animals recorded through whole-brain imaging. Each row is a neuron; neurons with fewer than four recordings in either genotype were excluded from analyses (rows shaded black). From left to right, column variables are as follows: (1) neuron *n*. (2) Overall dynamic range for that neuron (calculated as the s.d. of *F*/*F*_mean_ activity). Higher values show that this neuron’s activity changes more during a recording. From top to bottom, *P* = 0.0004, <0.0001, <0.0001. (3) Forward encoding strength (the slope of this neuron’s tuning to velocity, as defined in ref. ^15^). Positive values show a neuron is forward encoding, negative values indicate reverse encoding. All significant *P* = <0.0001. (4) Activity at the reversal end and during the postreversal turn. This is calculated as the average activity in the ~5 s around the ends of reversals followed by ventral turns. All significant *P* = <0.0001. (5) Median half-decay time or encoding timescale (as in ref. ^15^). Color bar is on a log scale. From top to bottom, *P* = <0.0001, 0.0004. (6) Expression levels of the five tyramine receptors (data from ref. ^48^). Black indicates that receptor expression is not detected. Differences in each category between WT and *tdc-1* were determined through a two-sided Wilcoxon rank-sum test with Bonferroni correction. **b**, Quantification of behavior in WT and *tdc-1(n3419)* animals during whole-brain imaging. Two-sided Wilcoxon rank-sum test with Bonferroni correction (from left to right, *P* = 0.025, 0.014, 0.0034). *n* = 17 *tdc-1* and 32 WT recordings. Data show mean ± s.e.m. **c**, Activity of forward-associated neurons in WT and *tdc-1* animals aligned to forward run starts. Dashed black line shows at run start; red shading shows the reversal. *n* = 275–762 runs (*n* values on the plot show the number of recordings with data for that neuron). Wilcoxon rank-sum test with Bonferroni correction comparing activity between genotypes during the run (black asterisks) and reversal (red asterisks; *P* = <0.0001, <0.0001, 0.0003, <0.0001, 0.0021). Data show mean ± 95% CI. **d**, Aligned activity of head-steering circuit neurons at reversal ends, as in Fig. 2b. Only reversals followed by ventral turns are shown. *n* = 328–523 reorientations (*n* values on the plot show the number of recordings per genotype with data for that neuron). Two-sided Wilcoxon rank-sum test with Bonferroni correction, comparing ~5 s (one head swing) before or after the reversal end (*P* = <0.0001, <0.0001, <0.0001, <0.0001). Data are mean ± 95% CI. **e**, *Z*-scored neuron activity aligned to head curvature, as in Fig. 2a. As head-curvature frequencies vary across time and animals, activity is aligned to the crossing from dorsal (positive) to ventral (negative) and vice versa, as in Fig. 2a. Head curvature for both genotypes is shown on the right. *n* = 329–468 time windows of forward movement, *n* = 82–130 time windows of reverse movement (*n* values on the plot show the number of recordings per genotype with data for that neuron). Data are mean ± 95% CI. **f**, Connectivity of RIM and the head-steering network, using data from ref. ^12^. **g**, Mock traces of RIM and the neurons of the head-steering network (reorientations in red), showing each neuron’s stereotyped changes across the behavior. These traces are drawn based on actual traces shown in Extended Data Fig. 8b. The first column lists behavioral features that we have shown affect each neuron’s activity. The second column shows the behavioral features we have shown are affected when these neurons are manipulated, either through optogenetics or cell-silencing/ablation experiments. For all panels, significance is noted as: NS (not significant), *P < 0.05, **P < 0.01, ***P < 0.001 and **P < 0.0001. For panels with multiple comparisons, symbols denote Bonferroni-adjusted *P* values.

The above results suggest that tyramine likely exerts its impact on navigation through multiple receptor types, suggesting widespread effects. Therefore, to examine the effects of tyramine at a brain-wide scale, we collected whole-brain calcium imaging datasets from 17 animals in a *tdc-1* mutant background (Fig. 7a,b). In these brain-wide recordings, the *tdc-1* mutants had the expected behavioral deficits—they exhibited slower, shorter, smaller angle reversals than WT animals (Fig. 7b).

**Extended Data Fig. 7:**
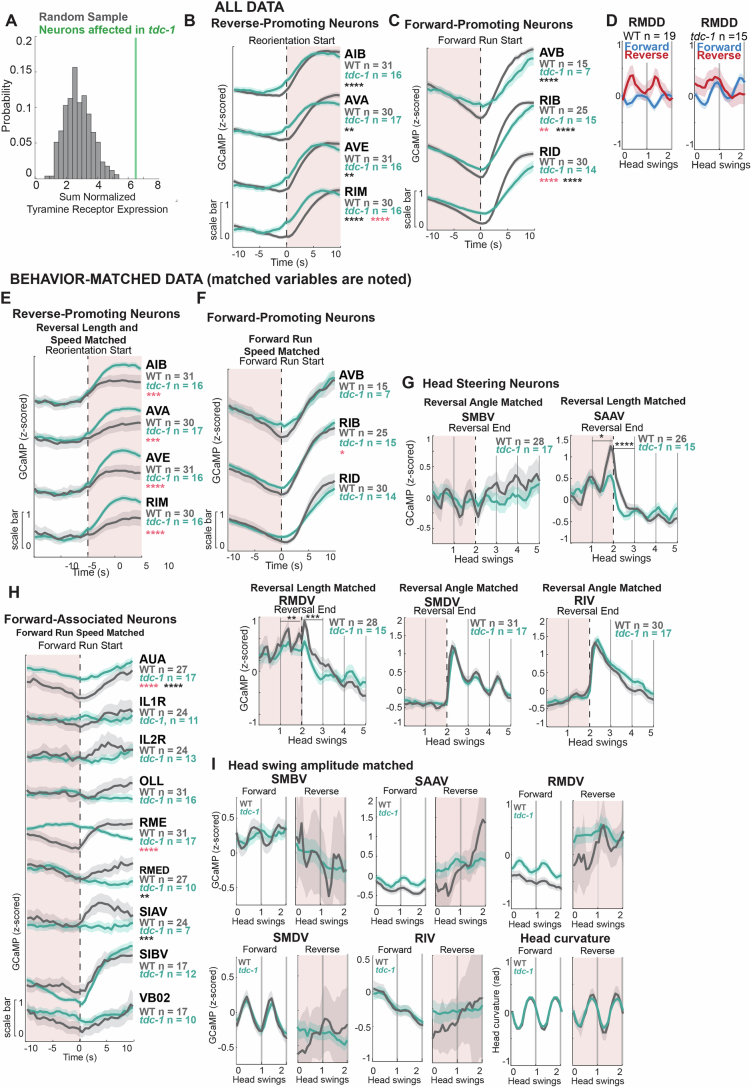
Further analysis of brain-wide activity changes in tyramine-deficient *tdc-1* mutant animals. **a**, Tyramine receptor expression is enriched in neurons that have significantly different encoding of behavior in *tdc-1* animals compared to wild-type animals. Gene expression data are from ref. ^48^, significant changes in encoding are shown and described in Fig. 7a. To compare tyramine receptor expression, expression for each neuron was normalized to the maximum transcripts per million (TPM) reported in any single neuron for that receptor, resulting in values ranging from 0 (no expression) to 1 (maximum relative expression). For example, *ser-2* is expressed most highly in OLL, at a TPM of 1104. The neuron NSM expresses *ser-2* at 170 TPM, so NSM ser-2 expression is normalized to a value of 0.15. We then compared the sum of expression across all tyramine receptors for all neurons that had altered encoding of behavior in *tdc-1* animals (both locomotion encoding, as reported in Fig. 7a, and head curvature encoding, defined as the slope of this neuron’s tuning to head curvature, using methods in ref. ^15^). The green vertical line shows the sum of the normalized tyramine receptor expression for the neurons that show significantly different locomotion and head curvature encodings between wild type and *tdc-1* backgrounds. The gray distribution shows the sum of normalized tyramine receptor expression for 500 randomly drawn sets of neurons (same size group of neurons as the real case). This analysis reveals that neurons that show changes in behavior encodings in *tdc-1* animals are significantly more likely to express tyramine receptors than randomly selected neurons, as the real data are at the 99th percentile of the randomly drawn distribution. **b**, Average activity of reverse-promoting neurons in all data for wild type and *tdc-1* animals, shown aligned to reorientation starts. Dashed black line shows reversal start; red shading shows the reversal. n = 570–762 reversals. n values on the plot show the number of recordings per genotype with data for a specific neuron. Two-sided Wilcoxon rank-sum test with Bonferroni correction comparing activity between genotypes, both during the run (black asterisks) and reversal (red asterisks). From top to bottom, *p* = <0.0001, 0.0001, <0.0001, <0.0001. Data show mean ± 95% CI. **c**, Average activity of forward-promoting neurons in all data from wild type and *tdc-1* animals, shown aligned to forward run starts. Dashed black line shows run start; red shading shows the reversal. n = 218–719 runs. Two-sided Wilcoxon rank-sum test with Bonferroni correction comparing activity between genotypes both during the run (black asterisks) and reversal (red asterisks). From top to bottom, *p* = <0.0001, 0.0002, <0.0001, <0.0001, <0.0001. Data show mean ± 95% CI. **d**, Z-scored RMDD activity aligned to head curvature (as in Fig. 2a) during forward (blue) or reverse movement (red). Left, wild-type data. Right, *tdc-1*. n = 112–447 time intervals of data during either forward or reverse movement. Data are mean ± 95% CI. As *tdc-1* animals’ behavior differs from wild type, and as we know behavior can affect neuron activity (for example, consider how SMDV activity scales with turn angle in Fig. 2c), we wanted to compare neuron activity in wild type versus *tdc-1* animals during similar behaviors. This could allow us to determine whether the relationship between activity and behavior was disrupted in *tdc-1* mutants per se. Therefore, **e**–**i** show neuron activity in *tdc-1* and wild-type animals during matched behaviors only. This was achieved by taking a subset of the data from either wild type or both wild type and *tdc-1* (indicated in **e**–**i**, each) and ensuring that the underlying behaviors were matched for relevant metrics as follows: reversal length, reversal speed, turn angle, or forward run speed. Different variables are controlled for different neurons—the exact variables controlled are determined based on neurons’ activities in wild type animals and are specified in this figure. (For example, SAAV activity changes based on reversal length in WT animals, so reversal length is matched for WT and *tdc-1* animals when looking at SAAV activity here.) **e**, Average activity of reverse-promoting neurons, aligned to reorientation starts, in reversal-matched wild-type and *tdc-1* animals. Wild-type data are limited to activity during reversals with a similar length and speed to *tdc-1* reversals. Dashed black line shows reversal start; red shading shows the reversal. n = 136–641 reversals. Two-sided Wilcoxon rank-sum test with Bonferroni correction comparing activity between genotypes, both during the run (black asterisks) and reversal (red asterisks). From top to bottom, *p* = <0.0001, <0.0001, <0.0001, <0.0001. Data show mean ± 95% CI. **f**, Activity of forward-promoting neurons, aligned to forward run starts, in matched wild-type and *tdc-1* animals. Wild-type and *tdc-1* data are limited to neuron activity during forward runs with similar speeds. Dashed black line shows run start; red shading shows the reversal. n = 77–441 runs. Two-sided Wilcoxon rank-sum test with Bonferroni correction comparing activity between genotypes, both during the run and reversal (*p* = 0.0011). Data show mean ± 95% CI. **g**, Z-scored neuron activity in neurons of the head-steering circuit at reversal ends (dashed black line), after which animals turn and resume forward movement. Neural data were aligned to a uniform head curvature frequency to preserve head curvature-associated neuron dynamics (Fig. 2a,b). Only reversals followed by ventral turns are shown. Wild-type data are limited to reversals of a similar length (SAAV, RMDV) or turn angle (SMBV, SMDV, RIV) to *tdc-1* animals. Matching metrics were chosen based on how these neurons respond to these behavioral metrics in wild-type animals. n = 176–373 reorientations. Two-sided Wilcoxon rank-sum test with Bonferroni correction, comparing one head swing before or after the reversal end (*p* = 0.001, <0.0001, 0.0006, <0.0001). Data are mean ± 95% CI. **h**, Activity of forward-associated neurons, aligned to forward run starts, in matched wild-type and *tdc-1* animals. Wild-type and *tdc-1* data are limited to forward runs with a similar speed. Dashed black line shows run start; red shading shows the reversal. n = 75–513 runs. Two-sided Wilcoxon rank-sum test Bonferroni correction comparing activity between genotypes, both during the run and reversal (from top to bottom, *p* ≤ 0.0001, <0.0001, <0.0001, 0.0001, <0.0001). Data show mean ± 95% CI. **i**, Z-scored neuron activity aligned to head curvature during forward (left panel) or reverse movement (right, shaded red). Neural activity was aligned to head curvature as in Fig. 2a. As *tdc-1* animals have lower amplitude head curvature (Fig. 7e), wild-type data here are limited to time points with similar amplitude head swings to *tdc-1* animals. This criterion resulted in very little wild-type data during reversals, hence the large confidence intervals. n = 13–411 time windows (each of two head swings). Data are mean ± 95% CI. For all panels, significance is noted as: NS (not significant), *P < 0.05, **P < 0.01, ***P < 0.001 and **P < 0.0001. For panels with multiple comparisons, symbols denote Bonferroni-adjusted *P* values.

We first surveyed whether the relationship between neural activity and locomotion behavior^15^ was disrupted in *tdc-1* mutants. This revealed that *tdc-1* mutants had dysregulated encodings and activities in many neurons of interest, including SAAV, RME and AUA (Fig. 7a). Comparing these results to the expression of tyramine receptors (Fig. 7a) revealed that neurons whose encodings of behavior were altered in *tdc-1* animals were significantly more likely to express tyramine receptors than expected by chance (Extended Data Fig. 7a). Although we note the majority of neurons that express tyramine receptors did not have significantly altered dynamics in *tdc-1* mutants, suggesting tyraminergic modulation may have context-specific effects.

We next examined the specific changes in neural activity during reorientation behaviors. (Results reported here are for all data; Extended Data Fig. 7e–i shows behavior-matched traces for WT and *tdc-1*, controlling for differences in behavior.) Activity of the reverse-promoting neurons (AVA, AVE, AIB and RIM itself) increased in the absence of tyramine (Extended Data Fig. 7b,e), but activity of the well-studied forward-promoting neurons (AVB, RIB and RID) was largely intact (Extended Data Fig. 7c,f). Marked deficits were seen in the other forward-active neurons characterized above—SIAV, AUA, RME(L/R) and RMED activity changes across forward-reverse transitions were essentially abolished in *tdc-1* (Fig. 7c and Extended Data Fig. 7h). As several of these neurons have a causal role in forward-reverse transitions (Fig. 4c,d), dysregulation of these neural dynamics may underlie part of the reorientation deficit in *tdc-1* mutants.

**Extended Data Fig. 8:**
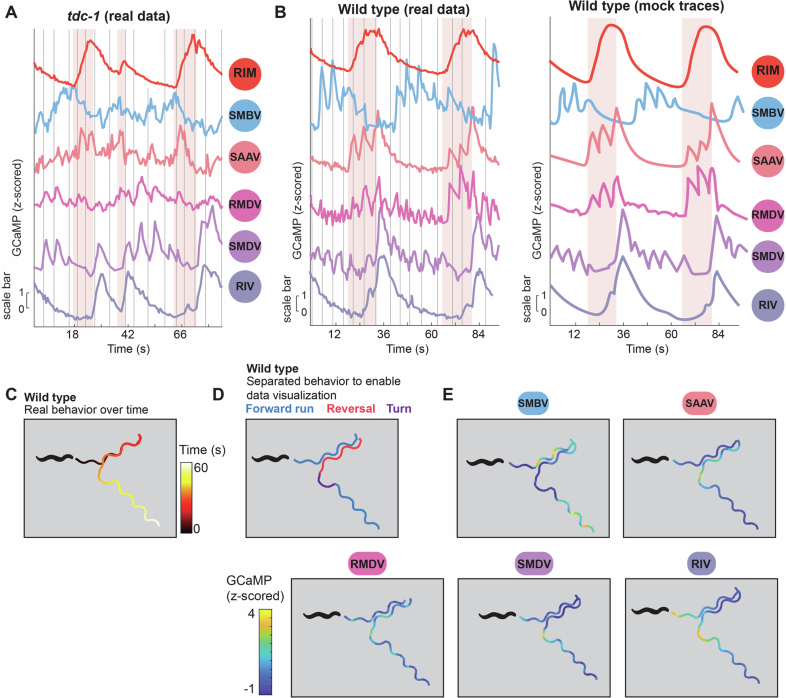
Example neural traces. **a**, Traces of RIM and the neurons of the head-steering network in an individual *tdc-1* animal across three reversals (shaded in red). Gray vertical lines show head curvature crossings from dorsal to ventral. These traces can be compared to the same neurons in a single wild-type animal (**b**). Similar to when data are pooled across animals (Fig. 7d,e and Extended Data Fig. 7g,i), responses in SMDV and RIV are largely unaffected in *tdc-1* animals compared to wild type, while SAAV, RMDV, and SMBV activities are dysregulated in *a tdc-1* background. **b**, Calcium traces of RIM and the neurons of the head-steering network in a wild-type animal across two reversals (shaded in red), showing each neuron’s stereotyped, sequential responses across each reorientation. Gray vertical lines show head curvature crossings from dorsal to ventral. Left, real traces of each of the neurons, which were recorded simultaneously in the same animal. Right, stylized mock traces for each neuron, which are presented in Figs. 2 and 7 as well. Mock traces were drawn based on the actual data to the left. **c**, The x/y position of the wild type animal in **b** over time. The trace specifically follows the movement of the animal’s head by tracking a point in the pharynx halfway between the metacorpus and grinder. In our brain-wide imaging conditions, we found that animals tend to trace over their forward movement during reverse movement, using the postreversal turn exclusively to change their direction, as can be seen here. **d**, The behaviors executed by the animal profiled in **b** and **c**, visually separating the forward and reverse movement by moving the path of reverse movement a few pixels away. This same separation is used in part (**e**). This separation is simply to assist visualization of activity overlays in **e**. **e**, Neuron activity for the head-steering network neurons layered onto the path of the animal’s motion (for the same animal in **b**–**d**). The x/y path of the animal is colored by the simultaneous z-scored neuron activity, showing how these neurons are active based on head position and movement phase. Again, reverse movement is slightly offset as in part (**d**) so that activity can be seen throughout the different phases of movement.

We also investigated neuronal activity in the head-steering circuit (Extended Data Fig. 7g,i shows behavior-matched data). Activity associated with forward head swings was largely unaltered in *tdc-1* animals (Fig. 7d,e). However, the activity of the neural sequence during reorientations was impaired in multiple neuron classes in *tdc-1* mutants, including SMBV, SAAV, RMDV and RMDD (Fig. 7d and Extended Data Fig. 7d,g,i). These changes are evident in example traces from WT and *tdc-1* animals (Extended Data Fig. 8a,b).

Disrupted activity across the head-steering circuit may underlie the impaired sensory-guided reorientation behaviors in *tdc-1* mutants. Notably, two of the neurons we had identified as having sensory-responsive activity, SAAV and SIAV (Figs. 2e–g and 4f,h), had dysregulated dynamics in *tdc-1* animals. More broadly, our *tdc-1* whole-brain calcium imaging results show that tyramine signaling is required for intact dynamics in many circuit elements relevant to reorientations (Fig. 7f,g), suggesting a widespread modulatory effect on brain activity during reorientations.

## Discussion

During navigation, neural circuits must process sensory information to generate motor sequences that result in sensory-directed movement. *C. elegans* uses odor gradient information to direct the initiation and angles of its reorientations, improving its bearing in the gradient. Neural activity during each reorientation occurs as a stereotyped neural sequence, where cells activate with precise dynamics in a reliable order. These neurons have distinct roles—responding to spatial locations of odors, anticipating upcoming turn directions, encoding turn kinematics and driving transitions to the next locomotion state (Fig. 7g). Therefore, the neural sequence that unfolds over time binds together a set of neurons with key elements of the sensorimotor behavior. Tyraminergic neuromodulation has a critical role in organizing these sequential neural dynamics.

Olfactory navigation in *C. elegans* is commonly described as a ‘biased random walk’. Consistent with this description, we observed that reorientation initiation is biased based on the odor gradient, matching many studies^20,21,23^. However, we additionally found that the angles and directions of individual reorientations were not random but instead were modulated to improve the animal’s bearing in the odor gradient. It is worth noting that these findings are consistent with previous chemotaxis literature, which lacked the resolution needed to record the angles of individual reorientations^20^. The navigation strategy that *C. elegans* uses seems to depend on the sensory context, as navigation to different cues yields distinct behavioral strategies^22,26,29,57^.

Our finding that *C. elegans* modulates turn direction even after short runs or reversals raises the possibility that the relevant sensory information may be gathered over very short timescales, possibly dorsal–ventral head swings, as proposed in the earliest chemotaxis literature^58^. However, these results are also consistent with the animal continually surveying the odor gradient during reorientations, essentially stopping its turn at the right time. A concurrent study found that asymmetric optogenetic activation of sensory neurons during forward movement can generate D/V-directed reorientations after a time delay^59^, which is more consistent with the former model. Additionally, we found that SAA activity could predict upcoming turn angles, also raising the possibility that the angle is predetermined before the turn. Predictive turning signals have also been observed in *Drosophila* and zebrafish^60,61^, suggesting this type of coding may be highly conserved.

What is the neural implementation of the gradual bias and eventual execution of sensory-guided turns that we identified in Fig. 1? Our data and prior work are currently most consistent with the following hypothesized model. During forward movement, oscillations in SMB and SAA activity can be modulated by attractive or aversive sensory cues. As reversals begin, RIM tyramine signals to the head-steering circuit to allow SAA and RMD to ramp up activity and compute turn sign and amplitude, based on existing SAA and SMB activity. Depending on the desired turn direction, asymmetric SAA and RMD activity then activates SMDD or SMDV to execute a dorsal or ventral turn.

We found that these head-steering circuit dynamics evolve rapidly during each reorientation, resulting in a stereotyped sequence of neural activity that underlies directed turning (Extended Data Fig. 8c–e). Interestingly, tyraminergic modulation was critical for many neurons’ activity changes during reorientations, consistent with tyramine’s ability to signal diffusely through ‘broadcasting’^48^. This population-level modulation, which occurs during each reorientation in *C. elegans*, aligns neural dynamics to allow for sensory-guided motor actions.

## Methods

### *C. elegans* genetics and growth conditions

WT animals were *C. elegans* Bristol strain N2. Animals were kept on Nematode Growth Medium (NGM) agar plates containing *Escherichia* *coli* OP50. Growth plates were maintained at 22 °C and 40% humidity. All experiments were conducted on 1-day-old young adults. Crosses were genotyped by PCR and/or sequencing as appropriate. Transgenic animals were generated through CRISPR–Cas9 genome editing or plasmid DNA injection into the gonads of young adult hermaphrodite animals. Transgenic strains were validated through sequencing or the presence of a fluorescent co-injection marker.

### Plasmids and promoters

We generated strains for cell-specific neuron silencing and optogenetic activation. Promoters were validated through expression of green fluorescent protein (GFP) fluorophores in the neurons of interest. The following promoters were used for cell-specific expression: AUA (*Pceh-6 + Pflp-8*, intersectional Cre/Lox)^15^, RIM (*Peat-4* *+* *Ptdc-1*, intersectional Cre/Lox), RIV (*Plad-2* *+* *Psri-5*, intersectional Cre/Lox), RME (*Punc-25* *+* *Pvap-1*, intersectional Cre/Lox), SAA (*Plad-2* *+* *Punc-42*, intersectional Cre/Lox)^36^, SIA (*Pceh-17* *+* *Ppdf-1*, intersectional Cre/Lox), SMB (*Pflp-12s*)^36^ and SMD (*Plad-2* *+* *Pfkh-10*, intersectional Cre/Lox). Intersectional promoters with Cre/Lox used previously described plasmid backbones^62^.

### New alleles generated in this study

The conditional rescue allele of *tdc-1* was made through CRISPR–Cas9 genome editing. The region containing the sixth through tenth exons of the endogenous *tdc-1* gene was inverted and placed between two sets of dual loxP sites in the FLEX arrangement. Additionally, an inverted t2a-GFP sequence was added immediately before the native stop codon in exon 10, such that successful reversion of the gene would result in cell-specific fluorescence. We confirmed cell-specific GFP expression in RIM and RIC in animals expressing Cre under their promoters (*eat-4* for RIM; *tbh-1* for RIC).

We constructed a new allele of *lgc-39* using CRISPR–Cas9 editing. In an existing strain lacking all four tyramine receptors (QW833)^63^, we made the following 46 bp insertion in LGC-39 (inserted sequence in bold), introducing a frameshift to the gene: ATAAATGGG**CAAACGAGTAGTAAGTAAGTAGTAAGTAGTAGTAAGTGATAAGCTA**GCCAAACGG. Mutant strains were backcrossed 3× to mitigate off-target mutations.

### Chemotaxis assays

Chemotaxis assays were performed as previously described^31,64^. A total of 50–200 young adult hermaphrodite animals are washed off the growth plates with chemotaxis buffer. Animals are then washed thrice in chemotaxis buffer to remove residual bacteria. Two 1-μl drops of 1 M sodium azide are added to each end of the plate to arrest movement if animals arrive at the odor or control end of the plate. Two 1-μl drops of odor were added to one side of the plate, and two 1-μl drops of ethanol (the diluting agent) were added to the other side. Animals were washed onto the center of the plate and excess liquid was dried using a Kimwipe. Assays were run on square grid plates with 10 ml of chemotaxis agar; plates were poured the night before. Assays were run at 22 °C and 40% humidity in a humidity-controlled incubator. After 1 h, plates were moved to 4 °C to stop movement. Assays were scored after over 1 day, and fluorescent strains were scored under the fluorescence microscope. Experimenters scoring the assay were blinded whenever possible. Animals were scored as follows: the number of animals at the odor, at the ethanol and on other parts of the plate were quantified. Animals remaining at the starting position were excluded. (This is the case for very few animals; in our butanone recordings, there were 0–4 animals in the center, with an average of 1.1 animals at the center per plate, and, in our octanol recordings, there were between 0–3 and an average of 1 animal in the center.) Chemotaxis index was calculated as (number of odor − number of ethanol)/(total number of animals). Odor concentrations used are—1:1,000 butanone (Sigma-Aldrich, 360473), 1:1,000 diacetyl (Sigma-Aldrich, 11038), 1:10 nonanone (Sigma-Aldrich, W278505) and 1:10 octanol (Sigma-Aldrich, 472328). Concentrations for each odor were determined by previously established maximally attractive concentrations for butanone and diacetyl^31^, the standard concentration for nonanone avoidance assays (for example, refs. ^65,66^) and the same concentration for octanol, which is within the range of its known aversive concentrations^32^.

### Multiworm recordings

Multi-animal behavior recordings were used to quantify locomotion as previously described^67^ both during optogenetic stimulation and during chemotaxis. For recording chemotaxis, the assay was identical to the above, except that only one drop of odor or ethanol was added to each side of the plate. Additionally, the recording plates lacked grids. Animals were also staged as L4 animals the night before. No odor controls for each genotype were collected from recording plates without odors, ethanol or azide to quantify spontaneous movement absent any known sensory cues. A total of 20–100 animals were recorded per plate. All tested strains were recorded over 2 days. WT controls were always recorded the same day as the mutant strain(s) to which they are compared. No odor controls for each genotype were likewise recorded on the same day as their counterpart odor recording plates.

To quantify behavior in response to a sharp odor gradient, we constructed an arena similar to that used for our imaging experiments described in the ‘Whole-brain imaging’ section. Sharp odor boundary environments allowed the pooling of data from animals that were experiencing similar changes in odor concentration to examine concurrent changes in neural activity, inspired by past work with microfluidics^21,24^. We first cut a square of flat NGM agar (1.25 × 1.25 cm). We then poured hot NGM agar with 0.167% octanol (40 μl of octanol was added to 24 ml of liquid NGM agar) around this square. This created a sharp octanol gradient without a gap as the hot agar fused to the first, cool agar. For the no-octanol control, NGM agar was poured around a central NGM square. For both conditions, the agar was then placed on a glass slide, and five to ten animals were picked off the food and onto the center NGM square. Animals were recorded for 10 min.

Optogenetic experiments were conducted similarly to previously described approaches^68,69^. L4 animals were staged the night before onto NGM plates seeded the previous day with 200 μl of OP50 with or without 50 μM all-*trans*-retinal. Animals were then maintained in the dark until the assay (16–20 h later). The assay then continued as described above, either with or without odor, as indicated. Light exposure was reduced whenever possible while washing and staging animals for recording. All experiments contain data recorded over 2 days. All recordings were acquired using JAI SP-20000M-USB3 CMOS cameras (5,120 × 3,840, monochrome) paired with a Nikon Micro-NIKKOR 55 mm f/2.8 lens, and captured using Streampix software at 3 fps. Illumination was from IR LEDs (Metaphase). For both CoChR and GtACR2 recordings, light illumination was at 470 nm and at 20 µW mm^−^^2^ from a Mightex LED. All recordings were analyzed with custom-built MATLAB scripts^67^.

For the optogenetic experiments described in Figs. 3 and 4, light stimulation patterns were as follows. Strains expressing the light-activated cation CoChR channel were exposed to no light for an initial period of 5 min. Animals were then under blue light for 20 s, then no light for 3 min, then blue light for 20 s and so on for a 30-min total recording. For strains expressing the light-activated chloride channel GtACR2, animals were exposed to no light for an initial period of 5 min. Animals were then under blue light for 60 s, then no light for 3 min, then blue light for 60 s and so on for a 30-min total recording.

From these videos, animals were segmented and tracked using previously published and described code that follows each animal’s centroid movement and posture^67^. Here we describe some of the previously described features of this behavioral quantification package relevant to our study, to aid understanding. We also describe all of the new behavioral parameters that we computed to describe navigation in this work.

#### Previously described properties of the behavioral tracker relevant to this study

The starts and ends of reversals were determined by times when the absolute value of the animal’s angular speed was over 75° s^−1^ (angular speed is determined by movement of the animal’s centroid). The spikes in angular speed reflect changes in direction (forward to reverse and vice versa). When two spikes occur in close succession (<8.8 s apart), this was considered a reversal event. The start time of the reversal was then considered to be the first frame at which angular speed was >75° s^−1^ during the first spike in angular speed, and the end time was the last frame before angular speed went below 75° s^−1^ during the second spike in angular speed. We defined pirouettes as consecutive reorientations occurring less than 13 s apart.

Turn type (omega versus mid-angle versus low-angle) was determined by both the change in direction and the posture of the animal during the turn. The change in direction was as follows: omega turns are >135°, mid-angle reorientations have a turn of 40–135° and low-angle reorientations (basically a pure reversal) have a turn of 0–40°. Omegas must additionally show the characteristic posture, as defined by the eccentricity of the animal. Mid-angle turns also required a change in eccentricity, although not as dramatic as the omega threshold. Turns must be within 1.5 s of the reversal end to be considered part of the same reorientation. High-angle and mid-angle turns were considered over as soon as worm eccentricity (that is, worm shape) went above a quantitative threshold to indicate that the animal was no longer coiled. Low-angle reorientations were over at the end of the reversal.

#### Behavior quantification developed in this study (not in previous description of tracker)

##### Direction to odor (*θ*)

The odor’s position was manually defined by the user based on its location on that particular plate. This location was used to define the angle between the animal’s position on the plate and the odor position. This angle was defined with respect to a uniform coordinate system in the video, where 0 is ‘south’. For a visualization, this angle would be the difference between ‘south’ and the dashed line between the animal and the odor in Fig. 1b. We then calculated the animal’s direction of movement on the plate (the arrow in Fig. 1b). This direction trajectory at time ‘t’ was defined as the change in the animal’s position on the plate between time *t* and *t* + 2 s. We found that smoothing over 2 s helped to reduce the jitter associated with sinusoidal movement. The angle between this direction of movement and the uniform coordinate system of the plate was then calculated (similar to the previous description). Direction to odor is then calculated as the angle between the angle of the animal’s movement and the angle between the animal and the odor. This direction to odor angle is shown as *θ* in Fig. 1b.

##### Bearing to odor (cos(*θ*))

The bearing to odor was the cosine of the direction to the odor (*θ*), as defined above. Bearing to odor values of 1 indicate an animal is moving directly toward the odor; values of −1 mean they are moving away from the odor. For spontaneous no-odor data, bearing to odor is calculated relative to the location where the odor would be on an odor plate.

As the animal’s movement trajectory changes rapidly during reorientations, this value is not calculated during the reorientation itself, but rather is considered as the animal begins forward movement after the reorientation (for example, in Fig. 1k). Similarly, the value at reorientation start considers the direction the animal was moving before the animal began the reorientation (for example, consider Fig. 1c).

##### Reorientation angle (*∆θ*)

The change in angle that the animal executed during a reorientation was calculated as the angle between their trajectory at the start of the reorientation and their trajectory at the end of the reorientation. The trajectory was defined similarly to the description above, except the direction at the start of the reorientation was defined based on their change in position in the 1 s before the reorientation, and direction at the end of the reorientation was defined based on their change in the 1 s after the reorientation.

##### Turn toward or away from the odor

To determine if each reorientation turned the animal toward or away from the odor, as in Fig. 1e,f, we compared the angle between the animal and the odor at the start of the reorientation (*θ*, here called *θ*_START_) to the angle between the animal and the odor at the end of the reorientation (*θ*_END_). We then compared the magnitude of the angles. If |*θ*_START_| > |*θ*_END_|, the reorientation was marked as turning the animal toward the odor, and vice versa (so, if |*θ*_START_| < |*θ*_END_|, the reorientation was marked as turning the animal away from the odor). We then calculated the fraction of reorientations that turned the animal toward the odor on each recording plate.

##### Fraction reorientations in the correct direction

To determine if each reorientation turned the animal in the correct or incorrect direction, we compared the angle between the animal and the odor (*θ*) at the beginning of the reorientation with the angle that the animal actually turned (∆*θ*) (for example, as in Fig. 1g,h). If these angles had the same sign, we assigned this as a ‘correct’ turn. If the angles’ signs differed, this was an ‘incorrect’ turn (visualization in Fig. 1e). The fraction of reorientations that turned the animal correctly was then calculated for each plate. We also note that our multiworm tracker recordings lacked sufficient resolution to anatomically identify each animal’s dorsal or ventral side. (Some animals have their ventral side on their right to a human observer and some on their left.) Therefore, the metric that we quantified could perhaps most accurately be described as the animal’s clockwise or counterclockwise correctness. This indicates whether animals direct their turns in the correct dorsal or ventral direction relative to their initial direction toward the odor. Notably, we do not need to know whether they turned dorsally or ventrally to calculate this metric—we only need to know whether they turned in the correct direction. To score the sharp-boundary recordings, reorientations that occurred <1 body length from the octanol (or NGM) boundary were considered. A reorientation was scored as ‘correct’ if it turned the animal in the opposite direction from which they encountered the agar boundary. For example, if the animal encountered the octanol on its dorsal side, a correct reorientation would turn them ventrally, away from this aversive stimulus.

##### Weathervaning

Weathervaning, as in Extended Data Fig. 1b, was calculated as previously defined^23^. During forward movement, we calculated the animal’s direction to the odor (*θ*), as defined above. We then calculated the animal’s curving rate. This value is the change in the animal’s heading angle with respect to the coordinate system of the plate (described above) divided by their displacement over the next 1 s. The interpretation of this value is that it tells how much the direction of their run is changing (magnitude) and if their run is bending in a certain direction (value). Comparing the sign of the curving rate and the direction to the odor, therefore, tells whether the animal is bending their run toward or away from the odor. If the signs are the same (that is, both positive), then the animal is bending its run direction toward the odor source, and vice versa. We included data only from the first 10 s of forward runs, as we found that WT animals exhibited weathervaning behavior most strongly during this period. Frames in which the animal was moving at <0.04 mm s^−1^ were excluded, as these were considered pauses.

### Single-worm recordings

Recordings of individual animals were conducted and analyzed as previously described using custom-built tracking software and microscopes^67,68,70^. In contrast to the multiworm tracker described above, single-worm recordings allow for the quantification of an animal’s posture, body angles, and head movement, rather than following the centroid position in the multi-animal setup. Animals were imaged on 10 cm standard NGM agar plates in the presence of 1 μl of 1:1,000 diluted butanone for consistency with other optogenetic experiments. Optogenetic experiments were conducted similarly to the above; briefly, L4 animals were staged the night before onto NGM plates seeded the previous day with 200 μl of OP50 with or without 50 μM all-*trans*-retinal. Animals were maintained in the dark for 16–20 h. All experiments contain data recorded over 2 days. Light illumination was at 532 nm and at 20 µW mm^−^^2^. Light stimulation began 4 min into the recording, then the laser was on for 60 s and off for 60 s four times (thus, a total of four separate stimulations per animal). Stage position and animal tracking rely on LabVIEW software. All recordings were analyzed with custom-built R (v3.6.1) and R Studio (v1.2.1335) scripts^70^, and the R output data were further analyzed with custom MATLAB scripts.

### Whole-brain imaging

Imaging was conducted and analyzed as previously described^15,18,30^. Recordings were conducted using the transgenic strains SWF702 or SWF1088 that have pan-neuronal GCaMP and NeuroPAL, as well as *lite-1* and *gur-3* null mutations^15,17^. We also generated a whole-brain imaging strain in *a tdc-1* background, SWF1002. This strain was made by crossing SWF702 animals to MT13113. Attractive odor experiments were conducted in SWF1088, which is SWF702 backcrossed to MT21793 an additional 11 times.

Recording hardware was as previously described for the octanol and *tdc-1* recordings^15^. For the diacetyl recordings, the cameras were Hamamatsu Orca Fusion BTSCMOS, although all other aspects of the hardware were the same. We recorded unbinned data on the Orca Fusion cameras and performed 3 × 3 binning of pixel intensities post hoc, resulting in a final image dimension of 322 × 210 × 80, which is the same as with the previous cameras.

Our priority in creating an imaging arena was to make a reliable sensory environment that generated a robust response so that we could compare behavior across individual animals. To present animals with an aversive olfactory stimulus during imaging, we cut a square of flat agar NGM (0.5 × 0.5 cm). We then poured hot agar around this square, which was NGM agar with 0.167% octanol (40 μl of octanol was added to 24 ml of liquid NGM agar). This created a sharp octanol gradient, as shown in Extended Data Fig. 1n. The agar layers were flush together (without a gap) as the hot agar fused to the first, cool agar layer. Both agar layers were sandwiched between a glass coverslip and a glass slide to ensure uniform thickness. We used octanol as we found WT responses to octanol were slightly more robust and reliable (for example, compare octanol and nonanone responses in WT animals in Fig. 5a,e,g). To achieve an attractive odor boundary, NGM agar was used to form both the agar center and surroundings, and then a thin layer of 1:1,000 diacetyl diluted in water was applied to the agar boundary with a paintbrush. This created a narrow band of attractive odor that animals would move on and off, as we were worried about habituation to the attractive odors if animals remained on the odor for too long. Diacetyl was chosen rather than butanone due to the stronger behavioral effect (for example, compare Fig. 1h to Extended Data Fig. 1d), and because diacetyl is sensed by both AWA neurons (L and R), whereas butanone is only sensed by one neuron, AWC-ON^71^, and we wanted to increase the odds of capturing the odor-responsive neuron in our brain-wide imaging. AWA is also much easier to identify than AWC in the NeuroPAL labeling system. For both odor conditions, once the agar pads were solidified, 9 μl of M9 buffer was placed on top of the pad, and 4 μl of 80 μm microsphere beads in M9 buffer was placed at the corners of the agar pad to alleviate pressure from the coverslip on the worm. One-day-old adults were mounted on the central NGM square. Animals were imaged for 8–16 min. Whenever possible, WT and *tdc-1* recordings were collected on the same day.

For whole-brain imaging behavior quantifications, reversals were defined as periods of backwards velocity (for example, compare red highlights showing reversals to the velocity trace in Fig. 1o). Postreversal turns were defined based on the animal’s body bending as the average change in direction in the 12 frames (7.2 s) after a reversal end (the average amount of time before animals returned to the stereotyped postures associated with forward movement). Head-curvature quantification was defined based on the angle along the anterior spline of the animal, specifically the angle between the direction from the tip of the animal’s nose to 35.4 μm along their body and the direction between 35.4 μm and 61.9 μm along the spline, as previously described^15^. Animal encounters with the octanol or diacetyl gradient were scored manually after the recordings. The ventral or dorsal side of the animal was defined visually based on the animal’s anatomy (some animals have their ventral side on their right to a human observer and some on their left).

#### Improvement in processing pipeline

The published ANTSUN pipeline for whole-brain imaging data processing^15,18,30^ includes a neural network for head detection. For each time point of a recording, the network takes in the maximum intensity projection of each volume across the *z* direction and predicts the *x* and *y* coordinates of the anterior region of the animal’s head. We improved this as follows. The network was retrained by incorporating more training data. We also tuned augmentation parameters and re-engineered the loss function to weigh near-misses less heavily. When evaluated against expert human annotation, the retrained model achieved an accuracy of >97%. As worms generally maintain the same head orientation over hundreds of consecutive time points, we interpolated the rare instances (2–3%) where the network had subthreshold confidence for head position with the head position of the previous time point.

#### Improvement on the cropping algorithm

In previous work^15^, we rotated and cropped the shear-corrected confocal volume to a variable size based on a fluorescence threshold. Volumes in which the animal’s posture was highly curled would be more likely to contain neurons not usually within the field of view (for example, part of the tail could be in view), creating registration difficulties and often leading to those volumes being dropped from analysis. In this work, we trained a two-dimensional U-Net to recognize the head neurons in a maximum intensity projection of the shear-corrected volume and used this network to replace pixel columns outside of the animal’s head with noise. We then rotated and cropped the volume to a uniform size. Implementing this network enabled a greater percentage of volumes to be registered and their data included in analysis. We reprocessed previously benchmarked sparsely labeled *eat-4::*GFP and pan-neuronal–GFP datasets^15^ using the new cropping and head-detection algorithms. We found that misregistration rates (<1%) and signal-to-noise were comparable to our previous findings^15^.

### Decoding postreversal turn direction from SAAV activity

To test whether SAAV activity and/or behavior could predict upcoming turn directions, we trained RNN decoder models. The models were tasked with taking SAAV activity and/or head curvature during a reversal and categorizing the event as preceding a ‘ventral’ versus ‘dorsal’ postreversal turn. We included all reversals that were sufficiently long (at least 1.5 head swings). For all such reversals, we extracted four-frame-long stretches of data (neural activity and behavior were both head-curvature-aligned as in Fig. 2b) from the reversal. Data within these time stretches were provided as input to the RNN decoder models. One model was trained on both the SAAV activity and head curvature (that is, behavior) within these time stretches. A control model was trained on only head-curvature behavior (neuron activation was set to zero). Sampling four frame-long time stretches from within the reversal prevented the models from easily guessing the sign of the upcoming turn based on preceding behavior, as the time stretches were not time-locked to reversal endings (that is, the models could not guess the animal would turn dorsal because the time stretch ended with a ventral head swing). Instead, the models could only provide accurate decoding if SAAV activity and/or head curvature were different in general during the reversals preceding dorsal versus ventral turns, which was the hypothesis that we were aiming to test. We chose to use an RNN decoder (rather than a linear decoder) because an RNN model could conceivably learn about time-varying signals and the correspondence between SAAV activity and head curvature.

#### Splitting of data into training, validation and test sets for cross-validation

To reduce stochasticity and gain confidence in our results, we used a hierarchical cross-validation scheme that allowed us to evaluate many models trained on different permutations of our data, which were always evaluated on unseen testing data. In this scheme, the overall dataset (that is, all reversal turns) was partitioned into five rotated 80:20 train/test split permutations. Within each training partition, the dataset was further split into four rotating 75:25 train/validation split permutations. This gives 5 × 4 = 20 unique train/validation/test split variations with 60:20:20 ratios, respectively. This scheme allowed us to train four different models and compute the average performance of these models on the same withheld testing data segment, allowing us to reach a more reliable conclusion regarding decoding accuracy than would be the case relying on a single trained model per testing data segment.

#### Selection of RNN

We chose to use an RNN for this analysis based on a comparison of several models’ performance on a comparable decoding task from head-curvature-associated neuron activity. We chose to select models based on this distinct task so as not to bias our model selection based on which performed best on the SAAV decoding. Specifically, we examined which model was best able to decode an animal’s direction of locomotion (forward versus reverse) based on RMDD activity and head curvature. This task was based on the finding that RMDD neurons invert how their activity correlated with head bending during forward compared to reverse movement (Extended Data Fig. 7b). We compared the performance of a general linear model, a general additive model and an RNN, and tested performance on withheld data. The RNN performed best on this task, and so we chose to use it for the SAAV task in Fig. 2d.

#### Model training

Due to *C. elegans*’ intrinsic bias to turn ventrally more frequently^33^, over half of our data were ventral turning events. We therefore took multiple steps to remove ventral bias during training and validation. To remove ventral bias during network training, in each round of training, we took a random subset of ventral events equal to the number of dorsal events. This allows the model to eventually train on all ventral events in the training set over multiple epochs, improving generalizability. Additionally, for each training/validation/test data split, we ensured that dorsal versus ventral turns were represented at similar ratios (that is, in each split, they are present at the ratio in the overall full dataset). During each epoch of model training, the training data were randomly partitioned into batches of 16. The model was trained for 250 epochs with a learning rate of 1 × 10^−3^ using the ADAM optimizer^72^.

#### Model architecture

The model used was a simple RNN using a GRUCell^73^ at each iteration. To avoid overfitting, we applied dropout to the final hidden state from the RNN. To smooth out the gradients while training, we then applied layer normalization. The normalized result is passed through a single linear layer with bias and then through a sigmoid activation function. The input size to the RNN was the number of channels (two—one for SAAV activity and the other for behavior), the hidden size was 3, and the output was a single floating point value between 0 and 1. Our model was constructed and trained using JAX^74^ and the neural network extension Equinox^75^.

#### Model evaluation

The model loss was evaluated using binary cross-entropy. This allows us to capture the accuracy and confidence of our RNN in a single metric. Accuracy was calculated by taking the final output from the model—that is, the prediction of a dorsal turn (output ≥ 0.5) or ventral turn (output < 0.5)—and rounding to either 0 or 1 to arrive at a binary classification. This was then compared to the actual turn direction.

#### Test accuracy

Each test split retains ventral and dorsal turning events at the ratio that they existed in our raw data (that is, with more ventral examples, due to animals’ intrinsic turning bias). Again, we sought to avoid bias, so we computed model accuracy on all ventral events and dorsal events separately and then averaged these two values together to get a total accuracy. This was essential because, for example, a model that learns nothing could always output a guess of ventral turn for every test example. In such a case, this method would report chance-level accuracy (50%), as desired. The accuracy reported from each model was chosen from the epoch where validation loss was minimized. The reported accuracies were then averaged across all 20 models.

#### *P*-value calculation

To compare the RNN trained on SAAV activity and behavior versus that trained on behavior only, we used the following procedure. For each of these two models, we obtained bootstrapped samples of testing set data and computed overall test accuracy on each bootstrap sample (this accuracy again weighed ventral and dorsal turns equally, as described above). This gave rise to sampling distributions with confidence intervals. We then computed the probability that the difference between these two distributions was nonzero, that is, the probability that the testing performance of the two models is different. This probability is reported as an empirical *P* value in Fig. 2d.

### Comparing tyramine receptor expression patterns and brain-wide encoding deficits in *tdc-1* mutants

In one analysis (Extended Data Fig. 7a), we examined whether tyramine receptor expression patterns were at all predictive of which neuron types have significantly different encoding of behavior in *tdc-1* animals compared to WT animals. To do so, we compared cell-specific gene expression data from ref. ^48^ to changes in neuronal encoding that we uncovered (Fig. 7a). We devised a way to describe the overall level of tyramine receptor expression in each neuron. In our approach, expression of each receptor was normalized to the maximum transcripts per million (TPM) reported in any single neuron for that receptor, resulting in values ranging from 0 (no expression) to 1 (maximum relative expression). For example, *ser-2* is expressed most highly in OLL, at a TPM of 1,104. The neuron NSM expresses *ser-2* at 170 TPM, so NSM *ser-2* expression is normalized to a value of 0.15. We then took the sum of expression across all five tyramine receptors for each neuron to obtain its overall level of tyramine receptor expression. In our analysis, we then summed these values across the neurons that had altered encoding of behavior in *tdc-1* animals. We compared this actual sum to a distribution of normalized tyramine receptor expression for 500 randomly drawn sets of neurons. These results are reported in Extended Data Fig. 7a.

### Subsampling of whole-brain imaging data

As WT and *tdc-1* animals have differing behavioral outputs (for example, Fig. 7b), and as we know behavior can affect neuron activity (for example, consider how SMDV activity scales with turn angle in Fig. 2c), for some analyses, we wanted to compare neuron activity in WT versus *tdc-1* animals during similar behaviors. This could allow us to determine whether the relationship between activity and behavior was disrupted in *tdc-1* mutants per se. Therefore, we compared neuron activity in *tdc-1* and WT animals during matched behaviors only, which we obtained by subsampling. We note that, in all such cases, we also presented all data without any subsampling and in the Results section noted any instances where there was a difference in the conclusion when analyzing the data these two ways (all such plots are in Fig. 7 and Extended Data Fig. 7). Briefly, we obtained matched behaviors by taking a subset of the data from either WT or both WT and *tdc-1* and ensuring that the underlying behaviors were matched for relevant metrics as follows: reversal length, reversal speed, turn angle, forward run speed and amplitude of head bending. Different variables were controlled for different neurons—the exact variables controlled are determined by neurons’ activities in WT animals and are specified in the legend and the figure for Fig. 7 and Extended Data Fig. 7. (For example, SAAV activity changes based on reversal length in WT animals (Extended Data Fig. 2c), so reversal length is matched for WT and *tdc-1* animals when looking at SAAV activity in Extended Data Fig. 7g.)

To subsample behavior, we first determined the underlying distributions of a behavior for each genotype (for example, reversal speed). We then determined the quintile distribution of this behavior for *tdc-1* animals. We then identified all WT reversals that fell into the range of each quintile and further determined the fewest number of matching reversals per quintile. We then randomly took an equivalent number of reversals from each quintile, creating a new, subsampled distribution of WT reversals that matched the *tdc-1* distribution for the parameter of interest. To further illustrate this approach, when considering reversal speed, the 60th–80th percentiles of *tdc-1* reversals are 0.065–0.078 mm s^−1^. Only 30 WT reversals fell in this range of speeds. We therefore took these, as well as 30 random WT reversals from each of the four other quintiles of the *tdc-1* distribution, essentially constructing a new distribution of data whose values were well matched to *tdc-1*. We then evaluated neural activity across these behavior-matched WT and *tdc-1* mutant datasets.

### Statistics and reproducibility

The statistical tests used are provided in the figure legends of Figs. 1–7 and Extended Data Figs. 1–8, as is the *n* for each experiment, and the definitions of center and dispersion. Statistics were calculated using MATLAB 2022a or GraphPad Prism (v10; Prism was only for chemotaxis indices). All statistical tests were two-sided, unless otherwise stated. No statistical methods were used to predetermine sample sizes, but our sample sizes are similar to those reported in previous publications (for example, refs. ^15,18,69^). Some visualizations and analyses used throughout the paper are described in more detail here.

Statistics in Figs. 2a and 7a rely on determining the fraction of datasets where the neuronal encodings of dorsal and ventral head curvature or velocity were significant, based on methods described in ref. ^15^. Briefly, these encodings are determined by fitting each neuronal activity trace with the previously described CePNEM model, which is an encoding model that predicts neural activity as a function of measured worm behavioral variables (velocity, head curvature and feeding). In essence, this model can be used to assign a quantitative metric of how much each neuron’s activity varies with ongoing animal behavior. Each behavioral metric is tested against the most naive null hypothesis—a given neuron’s activity does not encode that behavior. Therefore, the calculated *P* value, if significant, indicates that this neuron does encode this behavior. More specifically, this model is fit with Bayesian inference, allowing to compute the posterior distributions of all its parameters. One of those parameters describes the neuron’s encoding of head curvature, and we can compute an empirical *P* value that this parameter is either positive (dorsal encoding) or negative (ventral encoding). Encodings of forward movement and reverse movement are calculated similarly. If the relevant *P* value is significant after multiple-hypothesis correction, we declare that the neural trace encodes dorsal or ventral head curvature as appropriate. This same process is repeated for each behavior and each neuron in each recording, and the fraction of significantly encoding recordings is then determined and reported here. See ref. ^15^ for more details and control analyses (shuffle controls, etc.) used to validate this approach.

When examining how neuron activity varies based on head curvature during forward or reverse (Fig. 2a), head-curvature-responsive signals were lost if all data were averaged together, as head-swing frequency varies across time and animals. Therefore, neuronal activity was uniformly compressed or stretched to a uniform head-swing frequency of 4.8 s per head-swing cycle. We determined this value as it is the average frequency exhibited by WT animals in our recordings. We aligned activity to this frequency by first quantifying the head-curvature frequency for each particular time interval (we considered half-cycles of head curvature, which is between when the head curvature crossed from dorsal to ventral and when it crossed from ventral to dorsal, and vice versa). Based on this observed frequency for this animal and time period, we then correspondingly stretch or compress neural activity data from the same time period so that the cell’s activity at distinct phases of head curvature is aligned to the uniform frequency. Data are aligned to the crossing from dorsal to ventral (when head curvature goes from positive to negative) and the crossing from ventral to dorsal. All graphs show two complete head-swing cycles, and the *x* axis therefore shows head swings rather than time. Head curvature itself is always plotted using the same alignment for each plot and can be seen on the right of each plot.

When visualizing the activity of the head-steering neurons during postreversal turns (Fig. 2b), we again wanted to preserve their head-curvature-associated dynamics. We used an alignment method similar to the one described above. Neuron activity is aligned at dorsal to ventral crossing, and at ventral to dorsal crossing, at a uniform frequency before and after the reversal end. Gray vertical lines show each full head-swing cycle, which lasts 4.8 s. Neuron activity from each reversal and subsequent run is compressed or stretched based on the actual head-curvature frequency exhibited by that animal to align with the uniform frequency. Figure 2b separates the postreversal turns by whether the animals turn dorsally or ventrally, defined by taking the average head angle of that animal one to two frames postreversal. (Please note that the frequency is uniform rather than the sign of the alignment—postreversal dorsal versus ventral turns inherently involve the animals bending their heads in opposite directions, as can be seen in the head curvature plot on the right.) Figure 2c uses this same alignment for ventral turns only, separating the reversals by whether they are followed by small or large angle turns, defined as the cumulative change in direction that the animal exhibits in the 7.2 s postreversal. Here we called low-angle turns less than 90° of cumulative change in direction (and high-angle turns above 90°), a value that was chosen to split the WT turn data roughly in half (Fig. 7b). For all such graphs, statistics compare the average neuron activity in each classification (for example, dorsal versus ventral turns, or WT versus *tdc-1* animals) during one head swing before and after reversal end.

### Reporting summary

Further information on research design is available in the Nature Portfolio Reporting Summary linked to this article.

## Online content

Any methods, additional references, Nature Portfolio reporting summaries, source data, extended data, supplementary information, acknowledgements, peer review information; details of author contributions and competing interests; and statements of data and code availability are available at 10.1038/s41593-026-02257-5.

## Supplementary information

Supplementary Information Supplementary Note (strain list and primer sequences).

Reporting Summary

Supplementary Video 1 An animal during a brain-wide recording, showing NIR recording of behavior. The animal executes a reversal and turn beforeresuming forward movement.

## Data Availability

All brain-wide imaging data from this study is available at the following Dryad link: 10.5061/dryad.8sf7m0cz2.

## Code availability

Original code used to decode upcoming D/V turn direction from SAAV activity is available on GitHub: https://github.com/flavell-lab/SAAV_Decoding.

The Python code used for post hoc binning of pixel intensities is available on GitHub: https://github.com/flavell-lab/preprocess_videos.

The code for retraining and evaluation of the ANTSUN model performance is available on GitHub: https://github.com/flavell-lab/head_detector_unet2d.
