# Supplementary material for: Neural sequences underlying directed turning in Caenorhabditis elegans
Source: Nat Neurosci. 2026 Apr 10;29(6):1408–24. doi: 10.1038/s41593-026-02257-5 (PMC13246447; doi:10.1038/s41593-026-02257-5)
Supplement: Supplementary file 1 — Supplementary Note (strain list and primer sequences). [file 41593_2026_2257_MOESM1_ESM.pdf]

nature neuroscience

Supplementary information

https://doi.org/10.1038/s41593-026-02257-5

Neural sequences underlying directed turning in *Caenorhabditis elegans*

In the format provided by the authors and unedited

Supplementary Information

Strain List

STRAIN NAME	GENOTYPE	SOURCE
N2	Wild-type Bristol N2	*Caenorhabditis* Genetics Center (CGC)
CX3937	*lim-4(ky403)*	CGC
CX7155	*ins-1(nr2091)*	Cho et al. (2016)[1]
CX13779	*nlp-5(tm2125)*	Marquina-Solis et al. (2024)[2]
HBR2317	*nlp-8(syb762)*	CGC
CX12722	*lgc-46(ok2949)*	López-Cruz et al. (2019)[3]
JN1071	*snet-1(pe1063)*	CGC
CB1416	*unc-86(e1416)*	CGC
CB1111	*cat-1(e1111)*	CGC
MT13952	*lgc-53(n4330)*	CGC
RB2030	*nlp-3(ok2688)*	CGC
MT13113	*tdc-1(n3419)*	CGC
MT15434	*tph-1 (mg280)*	CGC
RB1659	*acr-3(ok2049)*	CGC
SWF748	*acr-6(ok3117)*	This study
SWF948	*flvEx452[inx-1::unc-103::sl2gFP (25 ng/uL)) myo-2:::mCherry (5ng/uL)]*	This study
SWF825	*flvEx388[eat-4::nCre (5ng/uL); ser-2b::inverted(unc-103, GFP) (30ng/uL); myo-2::mCherry (5ng/uL)]*	This study
ZX966	*zxIs28[pflp-1(trc)::ICE; pmyo-2::mCherry]*	Oranth et al. (2018)[4]
MT7988	*bas-1(ad446)*	CGC
MT15620	*cat-2(n4547)*	CGC
CB1141	*cat-4(e1141)*	CGC

LSC32	*ckr-2 (tm3082)*	CGC
KJ461	*cng-1(jh111)*	CGC
PS8860	*dmsr-4(sy1545)*	CGC
LX702	*dop-2(vs105)*	CGC
MT6308	*eat-4(ky5)*	CGC
KP2018	*egl-21(n476)*	CGC
ZM8969	*flp-14(gk1055)*	CGC
CX16983	*gar-1(ok755)*	López-Cruz et al. (2019)[3]
CX16986	*gar-2(ok520)*	López-Cruz et al. (2019)[3]
VC657	*gar-3(gk305)*	CGC
CX12708	*ggr-2(lf62)*	CGC
KP4	*glr-1(n2461)*	CGC
CX12720	*glr-2(tm669)*	López-Cruz et al. (2019)[3]
FX03506	*glr-5(tm3506)*	National BioResource Project (NBRP)
ZG31	*hif-1(ia4)*	CGC
FX16937	*Y63G10A.6(tm5866)*	National BioResource Project (NBRP)
FX18538	*inx-1(tm3524)*	National BioResource Project (NBRP)
MT2123	*let-23(n1045)*	CGC
MT14678	*lgc-40(n4545)*	CGC
CB1417	*lin-3(e1417)*	CGC
VC228	*nlg-1(ok259)*	CGC
MT15951	*nlp-3(n4897)*	Bhatla et al. (2015)[5]
SG1	*nrx-1(ds1)*	CGC
CX10	*osm-9(ky10)*	CGC
CX15050	*kyEx4999[Ppdfr-1::acy-1(gf)-sl2-mCherry, Pmyo-2::mCherry]*	Hilbert et al. (2018)[6]
MH1090	*pnc-1(ku212)*	CGC
SWF703	*kyEx4268[mod-1::nCre(8 ng/uL); myo-2::mCherry(1 ng/uL); kyEx4499 = odr-*	This study

	2(2b)::inv[TeTx::sl2gFP](25 ng/uL); myo-3::mCherry (5ng/uL)]	
EAH268	*bruEx160[twk-3::casp-3(p17), twk-3::casp-3(p12), myo-2::dsRed]*	Guillermin et al. (2017)[7]
RB987	*sbt-1(ok901)*	CGC
OH9331	*ttx-3(ot358).*	CGC
HBR232	*aptf-1(tm3287)*	CGC
VM1846	*glr-3(ak57)*	Bhatla et al. (2015)[5]
MT10661	*tdc-1(n3420)*	Alkema et al. (2005)[8]
MT10549	*tdc-1(n3421)*	Alkema et al. (2005)[8]
PHX7124	*tdc-1(syb7124)*	This study
SWF1044	*tdc-1(syb7124); flvEx518[eat-4::cre (10ng/uL); myo-2::mCherry (5ng/uL)]*	This study
SWF1045	*tdc-1(syb7124); flvEx519[tbh-1::cre (65ng/uL); myo-2::mCherry (5ng/uL)]*	This study
SWF946	*flvEx448[glr-1::Cre (18ng/uL); tdc-1::inv(CoChR)::sl2gFP 30ng/uL); myo-2:::mCherry (5ng/uL)]*	This study
SWF950	*flvEx453[glr-1::Cre (18ng/uL); tdc-1::inv(Gt2)::sl2gFP 30ng/uL); myo-2:::mCherry (5ng/uL)]*	This study
CX11839	*tyra-3(ok325)*	CGC
QW42	*tyra-2(1815)*	Donnelly et al. (2013)[9]
CX11501	*lgc-55(tm2913)*	Jin et al. (2016)[10]
OH313	*ser-2(pk1357)*	CGC
SWF882	*lgc-39(flv9); lgc-55(tm2913); tyra-3(ok325) tyra-2(tm1846); ser-2(pk1357)*	This study
SWF1002	*tdc-1(n3419); lite-1(ce314); gur-3(ok2245); otIs670[NeuroPAL]; flvIs17[tag-168::NLS-GCaMP7f, gcy-28.d::NLStag-RFPt, ceh-36:NLS-tag-RFPt, inx-1::tag-RFPt, mod-1::tagRFPt, tph-1(short)::NLS-tag-RFPt, gcy-5::NLS-tag-RFPt, gcy-7::NLS-tag-RFPt]*	This study

SWF1095	*flvEx546[ceh-6::cre (25ng/uL); flp-8::inv(CoChR sl2 GFP) (25ng/uL); myo-2::mCherry (5ng/uL)*	This study
SWF1097	*flvEx547[vap-1::cre (50 ng/uL); unc-25::inv(CoChR sl2 GFP) (20 ng/uL), myo-2::mCherry (5ng/uL)*	This study
SWF702	*lite-1(ce314); gur-3(ok2245); otIs670[NeuroPAL]; flvIs17[tag-168::NLS-GCaMP7f, gcy-28.d::NLStag-RFPt, ceh-36:NLS-tag-RFPt, inx-1::tag-RFPt, mod-1::tagRFPt, tph-1(short)::NLS-tag-RFPt, gcy-5::NLS-tag-RFPt, gcy-7::NLS-tag-RFPt]*	Atanas et al. (2023)[11]
SWF1142	*flvEx573(lad-2::cre (30 ng/uL); fkh-10::inv(Gt2) (25 ng/uL); myo-2::mCherry (5 ng/uL))*	This study
SWF1141	*flvEx572(lad-2::cre (30 ng/uL); fkh-10::inv(CoChR) (25 ng/uL); myo-2::mCherry (5 ng/uL))*	This study
SWF1139	*flvEx570(lad-2::cre (40 ng/uL); unc-42::inv(Gt2); myo-2::mCherry (5ng/uL))*	This study
SWF1144	*flvEx575(lad-2::cre (40 ng/uL); unc-42::inv(CoChR); myo-2::mCherry (5ng/uL))*	This study
SWF1136	*flvEx567(flp-12(s)::CoChR (25 ng/uL); myo-2::mCherry (5ng/uL))*	This study
SWF1182	*flvEx593(flp-12(s)::Gt2 (25 ng/uL); myo-2::mCherry (5 ng/uL))*	This study
SWF1156	*flvEx584(lad-2::Gt2 (15 ng/uL); myo-2::mCherry (5 ng/uL))*	This study
SWF1161	*flvEx589(lad-2::cre (30 ng/uL); fkh-10::inv(unc-103(gof)) (25 ng/uL); myo-2::mCherry (5 ng/uL))*	This study
SWF1143	*flvEx574(ceh-17::cre (50 ng/uL); pdf-1::inv(CoChR) (50 ng/uL); myo-2::mCherry (5 ng/uL))*	This study
SWF1146	*flvEx576(ceh-17::cre (50 ng/uL); pdf-1::inv(Gt2) (50 ng/uL); myo-2::mCherry (5 ng/uL))*	This study
MT11374	*tbh-1(n3722)*	Alkema et al. (2005)[8]

SWF972	*flvEx464[tdc-1::Cre (25ng/uL); glr-1::inv(unc-103)::sl2gFP 30ng/uL); myo-2:::mCherry (5ng/uL)]*	This study
SWF688	*flvEx301[tbh-1::TeTx sl2::mCherry (80ng/ul), elt-2::GFP (5 ng/uL)*	This study
SWF1088	*lite-1(ce314); gur-3(ok2245); otIs670[NeuroPAL]; flvIs17[tag-168::NLS-GCaMP7f, gcy-28.d::NLStag-RFPt, ceh-36:NLS-tag-RFPt, inx-1::tag-RFPt, mod-1::tagRFPt, tph-1(short)::NLS-tag-RFPt, gcy-5::NLS-tag-RFPt, gcy-7::NLS-tag-RFPt]*	This study
SWF1324	*flvEx653[lad-2::cre (40ng/uL); sri-5::inv(unc-103) (30ng/uL); myo-2::mCherry (5ng/uL)]*	This study
SWF1140	*flvEx571(vap-1::cre (70ng/uL); unc-25::inv(Gt2) (30 ng/uL); myo-2::mCherry (5ng/uL))*	This study
SWF1094	*flvEx545[ceh-6::cre (25ng/uL); flp-8::inv(GtACR2 sl2 GFP) (25ng/uL); myo-3::mCherry (5ng/uL)*	This study
SWF1350	*flvEx669[lad-2::cre (40ng/uL); sri-5::inv(Gt2) (30ng/uL); myo-2::mCherry (5ng/uL)]*	This study

Supplementary References

1. Cho, C.E., Brueggemann, C., L'Etoile, N.D., and Bargmann, C.I. (2016). Parallel encoding of sensory history and behavioral preference during Caenorhabditis elegans olfactory learning. eLife *5*, e14000. https://doi.org/10.7554/eLife.14000.

2. Marquina-Solis, J., Feng, L., Vandewyer, E., Beets, I., Hawk, J., Colón-Ramos, D.A., Yu, J., Fox, B.W., Schroeder, F.C., and Bargmann, C.I. (2024). Antagonism between neuropeptides and monoamines in a distributed circuit for pathogen avoidance. Cell Rep. *43*, 114042. https://doi.org/10.1016/j.celrep.2024.114042.

3. López-Cruz, A., Sordillo, A., Pokala, N., Liu, Q., McGrath, P.T., and Bargmann, C.I. (2019). Parallel Multimodal Circuits Control an Innate Foraging Behavior. Neuron *102*, 407-419.e8. https://doi.org/10.1016/j.neuron.2019.01.053.

4. Oranth, A., Schultheis, C., Tolstenkov, O., Erbguth, K., Nagpal, J., Hain, D., Brauner, M., Wabnig, S., Steuer Costa, W., McWhirter, R.D., et al. (2018). Food Sensation Modulates Locomotion by Dopamine and Neuropeptide Signaling in a Distributed Neuronal Network. Neuron *100*, 1414-1428.e10. https://doi.org/10.1016/j.neuron.2018.10.024.

5. Bhatla, N., Droste, R., Sando, S.R., Huang, A., and Horvitz, H.R. (2015). Distinct Neural Circuits Control Rhythm Inhibition and Spitting by the Myogenic Pharynx of *C. elegans*. Curr. Biol. *25*, 2075–2089. https://doi.org/10.1016/j.cub.2015.06.052.

6. Hilbert, Z.A., and Kim, D.H. (2018). PDF-1 neuropeptide signaling regulates sexually dimorphic gene expression in shared sensory neurons of C. elegans. eLife *7*, e36547. https://doi.org/10.7554/eLife.36547.

7. Guillermin, M.L., Carrillo, M.A., and Hallem, E.A. (2017). A Single Set of Interneurons Drives Opposite Behaviors in *C. elegans*. Curr. Biol. *27*, 2630-2639.e6. https://doi.org/10.1016/j.cub.2017.07.023.

8. Alkema, M.J., Hunter-Ensor, M., Ringstad, N., and Horvitz, H.R. (2005). Tyramine Functions Independently of Octopamine in the *Caenorhabditis elegans* Nervous System. Neuron *46*, 247–260. https://doi.org/10.1016/j.neuron.2005.02.024.

9. Donnelly, J.L., Clark, C.M., Leifer, A.M., Pirri, J.K., Haburcak, M., Francis, M.M., Samuel, A.D.T., and Alkema, M.J. (2013). Monoaminergic Orchestration of Motor Programs in a Complex C. elegans Behavior. PLOS Biol. *11*, e1001529. https://doi.org/10.1371/journal.pbio.1001529.

10. Jin, X., Pokala, N., and Bargmann, C.I. (2016). Distinct Circuits for the Formation and Retrieval of an Imprinted Olfactory Memory. Cell *164*, 632–643. https://doi.org/10.1016/j.cell.2016.01.007.

11. Atanas, A.A., Kim, J., Wang, Z., Bueno, E., Becker, M., Kang, D., Park, J., Kramer, T.S., Wan, F.K., Baskoylu, S., et al. (2023). Brain-wide representations of behavior spanning multiple timescales and states in C. elegans. Cell. https://doi.org/10.1016/j.cell.2023.07.035.

Primer Sequences for Promoter Regions

fkh-10
Forward: CACACAACTAATACGACTTC
Reverse: TCTTCTGAAATTATTTATAATAGAC

flp-8
Forward: GTCAGAAACCCCGATTCAAAC
Reverse: TTTCTACTTGAAAAGTGTGGACTG

flp-12(s)
Forward: CCGGGATTCCTCTATCCTT
Reverse: TGTTTACTGAAAGTTCAGC

sri-5
Forward: CTTCTTTGCCTTCTTACGACG

Reverse: TTTTTTGGCTGGAAAAGCTG

vap-1
Forward: ACTTCGTCAAACCCAACTCCAAAC
Reverse: CTGTGAAAATGAACGCACGCCTG
