# Supplementary material for: Neural sequences underlying directed turning in Caenorhabditis elegans
Source: Nat Neurosci. 2026 Apr 10;29(6):1408–24. doi: 10.1038/s41593-026-02257-5 (PMC13246447; doi:10.1038/s41593-026-02257-5)
Supplement: Supplementary file 2 — Reporting Summary [file 41593_2026_2257_MOESM2_ESM.pdf]

nature portfolio

Corresponding author(s): Steven Flavell

Last updated by author(s): 02/23/2026

Reporting Summary

Nature Portfolio wishes to improve the reproducibility of the work that we publish. This form provides structure for consistency and transparency in reporting. For further information on Nature Portfolio policies, see our Editorial Policies and the Editorial Policy Checklist.

Statistics

For all statistical analyses, confirm that the following items are present in the figure legend, table legend, main text, or Methods section.

n/a	Confirmed	
☐	☒	The exact sample size (*n*) for each experimental group/condition, given as a discrete number and unit of measurement
☐	☒	A statement on whether measurements were taken from distinct samples or whether the same sample was measured repeatedly
☐	☒	The statistical test(s) used AND whether they are one- or two-sided<br>*Only common tests should be described solely by name; describe more complex techniques in the Methods section.*
☐	☒	A description of all covariates tested
☐	☒	A description of any assumptions or corrections, such as tests of normality and adjustment for multiple comparisons
☐	☒	A full description of the statistical parameters including central tendency (e.g. means) or other basic estimates (e.g. regression coefficient) AND variation (e.g. standard deviation) or associated estimates of uncertainty (e.g. confidence intervals)
☐	☒	For null hypothesis testing, the test statistic (e.g. *F*, *t*, *r*) with confidence intervals, effect sizes, degrees of freedom and *P* value noted<br>*Give P values as exact values whenever suitable.*
☒	☐	For Bayesian analysis, information on the choice of priors and Markov chain Monte Carlo settings
☒	☐	For hierarchical and complex designs, identification of the appropriate level for tests and full reporting of outcomes
☒	☐	Estimates of effect sizes (e.g. Cohen's *d*, Pearson's *r*), indicating how they were calculated

Our web collection on statistics for biologists contains articles on many of the points above.

Software and code

Policy information about availability of computer code

Data collection	The following commercial software was used: MATLAB (2022a) and Streampix (v7.0).
Data analysis	The following commercial software was used: MATLAB (2022a), R (v3.6.1), R Studio (v1.2.1335), and GraphPad Prism (v10). Custom code used for the analysis of neuron activity decoding, post-hoc pixel intensity binning, and ANTSUN model performance re-training and evaluation are available on the provided GitHub link.

For manuscripts utilizing custom algorithms or software that are central to the research but not yet described in published literature, software must be made available to editors and reviewers. We strongly encourage code deposition in a community repository (e.g. GitHub). See the Nature Portfolio guidelines for submitting code & software for further information.

Data

Policy information about availability of data

All manuscripts must include a data availability statement. This statement should provide the following information, where applicable:

- Accession codes, unique identifiers, or web links for publicly available datasets
- A description of any restrictions on data availability
- For clinical datasets or third party data, please ensure that the statement adheres to our policy

All brain-wide imaging data from this study is available at the following Dryad link: http://datadryad.org/share/L731GV6Ab6VebP9u4_jPxOkUCbFtk7tTrbLZrgP4BXU

Research involving human participants, their data, or biological material

Policy information about studies with human participants or human data. See also policy information about sex, gender (identity/presentation), and sexual orientation and race, ethnicity and racism.

Reporting on sex and gender	n/a
Reporting on race, ethnicity, or other socially relevant groupings	n/a
Population characteristics	n/a
Recruitment	n/a
Ethics oversight	n/a

Note that full information on the approval of the study protocol must also be provided in the manuscript.

Field-specific reporting

Please select the one below that is the best fit for your research. If you are not sure, read the appropriate sections before making your selection.

☒ Life sciences ☐ Behavioural & social sciences ☐ Ecological, evolutionary & environmental sciences

For a reference copy of the document with all sections, see nature.com/documents/nr-reporting-summary-flat.pdf

Life sciences study design

All studies must disclose on these points even when the disclosure is negative.

Sample size	Sample size was determined based on similar prior experiments in the field and our understanding of the amount of data needed to achieve reliable, believable results. For brain wide imaging, we sought to collect >20 wild type datasets across many days based on our past understanding of the variability of neuron activity across datasets (Atanas 2023). For behavioral experiments, we always recorded animals on >=2 days in order to account for day-to-day-variations in behavior. As the lab has the capacity to record up to 7 plates of animals at a time, this typically resulted in sample sizes of 10-20 plates across the 2-3 days.
Data exclusions	Data was not excluded.
Replication	Behavioral experiments were always conducted on >=2 days in order to ensure behavior replicated across days. Brain wide imaging data was collected on many days, and wild type and tdc-1 animals encountering octanol were imaged on the same day whenever possible.
Randomization	In cases where animals were tested across conditions (for example, placed in an arena with an odor versus a no odor control), animals were raised in identical conditions and then plates of animals were randomly assigned to be given odor or no odor.
Blinding	For any metric scored by an experimenter, the experimenter was blinded to the genotype of the animals and the identities of the odors (for example, for all scoring of chemotaxis assays in Fig 5). For all automated analysis of behavior and brain-wide activity (for example, Fig 1 navigation strategies), genotype and odor were not blinded, as the experimenter did not control the outcome of the analysis.

Reporting for specific materials, systems and methods

We require information from authors about some types of materials, experimental systems and methods used in many studies. Here, indicate whether each material, system or method listed is relevant to your study. If you are not sure if a list item applies to your research, read the appropriate section before selecting a response.

Materials & experimental systems

n/a	Involved in the study	
☒	☐	Antibodies
☒	☐	Eukaryotic cell lines
☒	☐	Palaeontology and archaeology
☐	☒	Animals and other organisms
☒	☐	Clinical data
☒	☐	Dual use research of concern
☒	☐	Plants

Methods

n/a	Involved in the study	
☒	☐	ChIP-seq
☒	☐	Flow cytometry
☒	☐	MRI-based neuroimaging

Animals and other research organisms

Policy information about studies involving animals; ARRIVE guidelines recommended for reporting animal research, and Sex and Gender in Research

Laboratory animals	Wild type animals were C. elegans Bristol strain N2. All other strains used are listed in Methods.
Wild animals	Study did not involve wild animals.
Reporting on sex	All animals are hermaphrodites.
Field-collected samples	Study does not involve field-collected samples.
Ethics oversight	No ethical guidance was required as C. elegans are invertebrates

Note that full information on the approval of the study protocol must also be provided in the manuscript.

Plants

Seed stocks	Plants were not used
Novel plant genotypes	Plants were not used
Authentication	Plants were not used
